# Cell-intrinsic and -extrinsic effects of SARS-CoV-2 RNA on pathogenesis: single-cell meta-analysis

**DOI:** 10.1128/msphere.00375-23

**Published:** 2023-09-22

**Authors:** Mst Shamima Khatun, T. Parks Remcho, Xuebin Qin, Jay K. Kolls

**Affiliations:** 1 Departments of Pediatrics & Medicine, Center for Translational Research in Infection and Inflammation, Tulane University School of Medicine, New Orleans, Louisiana, USA; 2 Tulane National Primate Research Center, Covington, Louisiana, USA; 3 Department of Immunology and Microbiology, Tulane University School of Medicine, New Orleans, Louisiana, USA; Johns Hopkins University Bloomberg School of Public Health, Baltimore, Maryland, USA

**Keywords:** COVID-19, animal models, single-cell RNA-seq, meta-analysis

## Abstract

Single-cell RNA-seq has been used to characterize human COVID-19. To determine if preclinical models successfully mimic the cell-intrinsic and -extrinsic effects of severe disease, we conducted a meta-analysis of single-cell data across five model species. To assess whether dissemination of viral RNA in lung cells tracks pathology and results in cell‐intrinsic and ‐extrinsic transcriptomic changes in COVID-19. We conducted a meta-analysis by analyzing six publicly available, scRNA-seq data sets. We used dual mapping (host and virus) and differential gene expression analyses to compare viral^+^ and viral^−^ cell populations. We conducted a principal component analysis to identify successful models of human COVID-19. We found expression of viral RNA in many non-epithelial cell types. Fibroblasts, macrophages, and endothelial cells exhibit clear evidence of viral-intrinsic and -extrinsic effects on host gene expression. Using viral RNA expression, we found that K18-hACE2 mice most closely modeled severe human COVID-19, followed by hamsters. Ferrets and macaques are poor models of human disease due to the low presence of viral RNA. Moreover, we found that increased transcripts of certain key inflammatory genes such as *IL1B, IL18*, and *CXCL10* are not restricted to virally infected cells, suggesting these genes are regulated in a paracrine or autocrine fashion. These data affirm widespread dissemination of viral RNA in the lung, which may be key in the pathogenesis of severe COVID-19 and demonstrate ferrets and Rhesus macaques are poor models of human COVID-19.

**IMPORTANCE:**

We conducted a high-resolution meta-analysis of scRNA-seq data from humans and five animal models of COVID-19. This study reports viral RNA dissemination in several cell types in human data as well as in some of the pre-clinical models. Using this metric, the K18-hACE2 mouse model, followed by the hamster model, most closely resembled human COVID-19. We observed clear evidence of viral-intrinsic effects within cells (e.g., *IRF5* expression) as well as viral-extrinsic cytokine modulation (e.g., *IL1B, IL18, CXCL10*). We observed proinflammatory chemokine expression in cells devoid of viral RNA expression, suggesting autocrine/paracrine interferon regulation. This report serves as a resource-synthesizing data from COVID-19 humans and animal models and suggesting improvements for relevant pre-clinical models that may aid future diagnostic and therapeutic development projects.

## INTRODUCTION

Severe acute respiratory syndrome coronavirus 2 (SARS-CoV-2) infection leads to coronavirus-induced disease (COVID-19), a critical threat to global healthcare systems (1). Human studies are limited by sampling timing relative to exposure, viral dose and route discrepancies, and limited sample availability for in-depth tissue analysis. These challenges are overcome by using animal models. Currently, COVID-19 is modeled in hamsters, K18-human ACE2 transgenic mice (K18-hACE2), African green monkeys (AGMs), ferrets, and Rhesus macaques (2). The K18-hACE2 model has been used to examine COVID-19 disease progression and therapeutic efficacy (3, 4). Studies indicate hamsters can be infected with SARS-CoV-2 and exhibit COVID-19 pathogenesis closely resembling human disease (5). Ferrets are a commonly used animal model of respiratory virus pathogenesis (6, 7).

Recently, single-cell RNA sequencing (scRNA-seq) studies have been published for these models using whole lung or, in humans, cells from bronchoalveolar lavage fluid (BALF) (4, 8). Although scRNA-seq has been used to characterize host inflammatory responses, the role of cell-intrinsic viral RNA using a dual-sequence reference methodology has not been thoroughly explored. Moreover, the field lacks a current meta-analysis of these data sets. Thus, we conducted a comprehensive analysis of scRNA-seq data from humans and all five animal models. This analysis revealed viral RNA dissemination in several cell types. In agreement with prior data, we show that in K18-hACE2, viral RNA is present in non-epithelial lung cells (9). We found that K18-hACE2 most closely models human COVID-19. We observed viral transcriptomic differences in several cell types with clear evidence of viral-intrinsic effects (e.g., *IRF5* expression) and viral-extrinsic effects (e.g., proinflammatory cytokines/chemokines: *IL1B, IL18, CXCL10*) (10). We observed proinflammatory chemokine expression in cells devoid of viral RNA expression, suggesting autocrine/paracrine interferon regulation.

## MATERIALS AND METHODS

### Data sources

We analyzed six publicly available scRNA-seq data sets (raw fastq files) from critical human patients and five animal models (Table 1). For K18-hACE2 (9), samples from 4-day post-SARS-CoV-2 infection and a healthy control whole lung were downloaded from the GSE (Genomic Spatial Event) database under accession number GSE175996 (11) and two samples from 48 hours post-SARS-CoV-2 infection whole lung were deposited under accession number GSE239835. Syrian hamster whole-lung data sets were downloaded from GEO (Gene Expression Omnibus) under accession number GSE162208 (5). For human patients, we downloaded BALF data from six patients (12) with severe/critical infection (S1–S6) (Table S1) and 10 healthy controls (13) from GEO under accession numbers GSE145926 and GSE151928, respectively. The AGM data were downloaded from a published study, GSE156755 (14). Ferret data files were retrieved using GSE171828 (7). The Rhesus macaques’ data set was also downloaded from a published study, GSE190659 (8). Additional details on the method for dual-custom references, processing of scRNA-seq data (including ambient RNA exclusion), marker detection and differential expression analysis, Venn diagram, PCA and line chart, and IGV analysis are provided in an online data supplement.

**TABLE 1:** Human patients and animal models information for single-cell RNA-seq analysis from six different species^*a*^

Human and animal	Species	Gender	Inoculation		Total samples	Virus strain	Tissue and cells	GEO	References
			Dosage	Route					
K18-hACE2 mice	*Mus musculus*	Female	2 x 105 TCID50	IN	1–4 dpi 2–48 h	USA-WA1/2020	Lung	GSE175996, GSE239835	(9)
Syrian Hamsters	*Mesocricetus auratus*	Female and male	1 × 105 pfu SARS- 759 CoV-2	IN	3–3 dpi 3-ctrl	BetaCoV/ Germany/ BavPat1/ 2020	Lung	GSE162208	(5)
Human	*Homo sapiens*	Male and female	NA	NA	6-inf 10-ctrl	Wu	BALF cells, BALF cells	GSE145926, GSE151928	(12, 13)
African Green Monkey	*Chlorocebus aethiops*	Male and female	4 × 10 TCID50	IN, IT, O, OC	4–3 dpi 4–10 dpi 2-ctrl	nCoV-WA1-2020	BALF cells	GSE156755	(14)
Ferrets	*Mustela putorius furo*	Male and female	10^5.5^ TCID_50_	IN	3–2 dpi 4–5 dpi 3-ctrl	NMC2019-nCoV02 virus	BALF cells	GSE171828	(7)
Rhesus Macaques	*Macaca mulatta*	NA	1.05 × 106 pfu SARS- CoV-2	IN, IT, OC	6–3 dpi 6-ctrl	USA-WA1/2020	BALF cells	GSE190659	(8)

^*a*^ K18-hACE2 = a transgenic mouse strain expressing human angiotensin-converting enzyme 2 (hACE2) under the K18 promoter; SARS-CoV-2 = severe acute respiratory syndrome coronavirus 2; TCID = tissue culture infectious dose; pfu = plaque-forming unit; IN = intranasally; IT = intratracheal; O = oral; OC = ocular; WA = Washington; Wu = Wuhan; dpi = day post infection; inf = infected; ctrl = control; NA = not available; BALF = bronchoalveolar lavage fluid; GEO = Gene Expression Omnibus.

## RESULTS

### Differential expression analysis in mouse and hamster lung tissue

**Fig 1:**
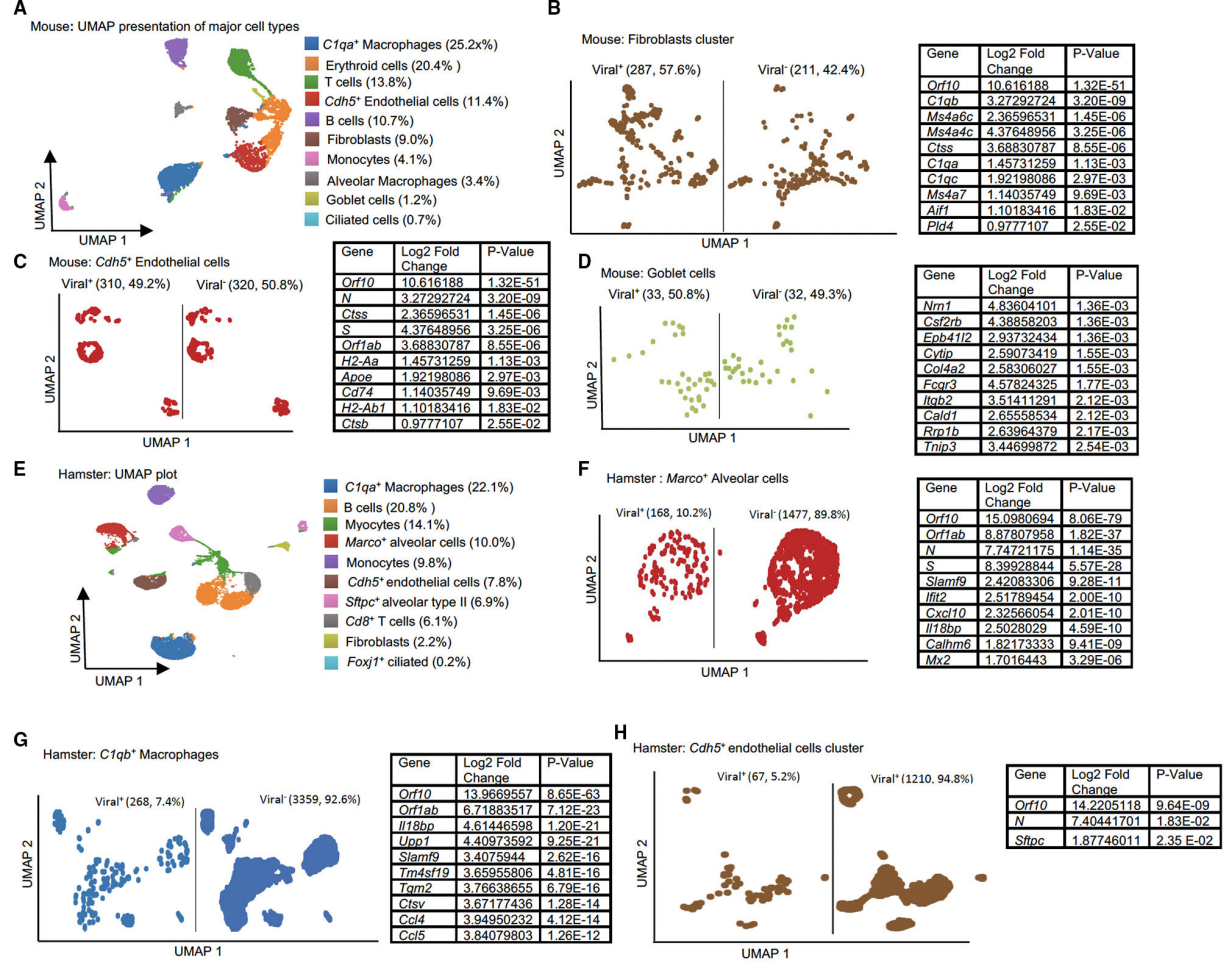
Single-cell RNA-seq analysis of COVID-19-infected mouse and hamster whole lungs identifies dissemination of viral RNA in diverse cell populations. All samples were analyzed using canonical correlation analysis within the Seurat R package and Loupe Browser (10x Genomics). Cells were clustered using a K-means clustering approach and visualized using a uniform manifold approximation and projection (UMAP) plot. Differentially expressed genes were analyzed by comparing viral^+^ (*Orf10*) versus viral^−^ in the same cluster. (**A**) Major clusters and respective cell-type features for mouse lungs. (**B–D**) UMAPs are shown for viral^+^ (*Orf10*) and viral^−^ cells and a table lists significant differentially expressed genes (upregulated in viral^+^ cells) in the (**B**) fibroblasts, (**C**) *Cdh5^+^* cells, and (**D**) T cells. (**E**) Cellular populations identified for hamster lungs. (**F–H**) Differential expression analysis of single-cell RNA-seq data from infected lungs identifies significant genes within (**F**) *Marco^+^* alveolar cells, (**G**) *C1qb^+^* macrophages, and (**H**) *Cdh5^+^* cells. A gene is considered significant with adjusted *P* < 0.05 (*P*-value adjusted by multiple testing in the Wilcoxon rank-sum test).

To investigate immune activation, cellular diversity, and viral gene expression in different models, we examined published whole-lung scRNA-seq data from SARS-CoV-2-infected K18-hACE2 (*Mus musculus*) (8) (Table 1). We visualized the data using dimensionality reduction (uniform manifold approximation and projection, UMAP). Resultant unsupervised analysis of 5,508 total cells revealed 10 major cell clusters, including *C1qa* ^+^ macrophages (25.2%), erythroid cells (20.4%), T cells (13.8%), *Cdh5* ^+^ endothelial cells (11.4%), B cells (10.7%), fibroblasts (9.0%), monocytes (4.1%), alveolar macrophages (3.4%), goblet cells (1.2%), and ciliated cells (0.7%) (Fig. 1A). In this data set, the mean reads per cell was 81,329 (Table S2). The most abundant viral RNA detected were *Orf10*. We found expression of *Orf10* in 10 distinct cell clusters (Table S3) with the greatest preponderance of *Orf10* in alveolar macrophages (90.43%), followed by *C1qa^+^* macrophages (83%). We also observed evidence of *Orf10* expression in 58% of fibroblasts, 49% of *Cdh5^+^* cells, 50% of goblet cells (Fig. 1B through D), 28% of T cells, 40% of erythroid cells, 24% of B cells, 39% of monocytes, and 55% of ciliated cells (Fig. S1A through G).

To understand how cell-intrinsic viral RNA affects the host transcriptome, we compared viral^+^ and viral^−^ cells within the same cluster and conducted differentially expressed gene (DEG) analysis. Despite relatively lower viral RNA dissemination, we found the greatest number of significant DEGs (sDEGs, 45 genes) in fibroblasts, including 41 host genes (e.g., *Ifi30, Il1rn, C1qa/b/c, Fcer1g, Cd14, Cd68*, *Ccl9*) and four viral transcripts (*Orf10, Orf1ab, N,* and *S)* (Fig. 1B; Table S4). In the *Cdh5* ^+^ endothelial cell cluster, we found 18 sDEGs, including 14 host genes (e.g., *H2-Aa, Cxcl2, Ctss*) and four viral RNAs (*Orf10, Orf1ab, N,* and *S*) (Fig. 1C; Table S5). Goblet cells also showed 32 sDEGs (31 host genes), including *Ccl6, Fcgr3,* and *Ctss. Orf10* was the only viral gene expressed in this cluster (Fig. 1D; Table S6). The T cell cluster showed 10 sDEGs, including six host genes (e.g., *H2-Aa*) and four viral transcripts (*Orf10, Orf1ab, S,* and *N*) (Fig. S1A). Despite the *C1qa* ^+^ macrophage cluster having a higher percentage of viral RNA^+^ cells, DEG analysis showed mainly viral genes (*Orf10, Orf1ab,* and *S*) were DE and only the host gene *Hpqd* was DE (Fig. S1B). Similar results were also observed in erythroid, B cell, and monocyte clusters, where we found viral sDEGs (*Orf10, N, Orf1ab,* and *S*) but no host sDEGs (Fig. S1C through E). Despite the presence of viral RNA in alveolar macrophages and ciliated cells, there were no DE host genes unique to viral^+^ cells (Fig. S1F and G). Importantly, the “presence of viral RNA” or “viral^+^ cells” may or may not be due to infection. This may be due to residual ambient RNA or cell-associated viral RNA or viral RNA that is packaged in exosomes (15).

We evaluated Syrian hamsters next (*Mesocricetus auratus*), using three samples from day 3 post-infection (Table 1). scRNA-seq was performed on the whole lung (5). Analysis of 16,382 cells revealed 10 clusters: *C1qb* ^+^ macrophages (22.1%), B cells (20.8%), myocytes (14.1%), *Marco* ^+^ alveolar cells (10.0%), monocytes (9.8%), *Cdh5* ^+^ endothelial cells (7.8%), *Sftpc* ^+^ alveolar type II cells (6.9%), *Cd8* ^+^ T cells (6.0%), fibroblasts (2.2%), and *Foxj1* ^+^ ciliated cells (0.2%) (Fig. 1E).

**Fig 2:**
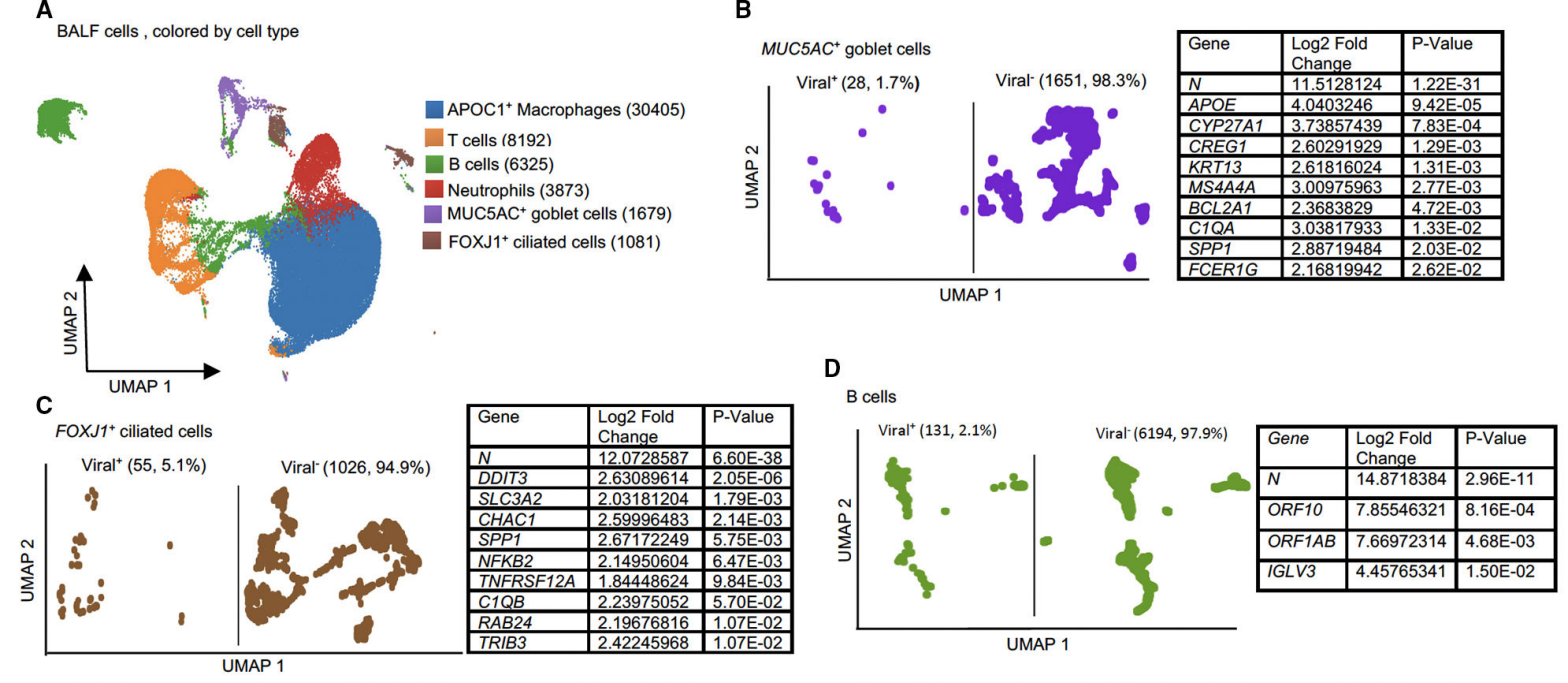
Dissemination of viral RNA in different cell populations of COVID-19-infected severe human patients BALF. (**A**) UMAP plot showing the clustering of 51,555 cells into six distinct cellular types. (**B–D**) Differential expression analysis of single-cell RNA-seq data from infected BALFs. UMAPs showing viral^+^ and viral^−^ cells within (**B**) *MUC5AC* ^+^ goblet cells, (**C**) *FOXJ1^+^* cells, and (**D**) B cells and tables showing the significant upregulated genes compared with viral^+^ and viral^−^ cells in the same cluster. Significancy is determined using an adjusted *P* < 0.05 (*P*-value adjusted by multiple testing in the Wilcoxon rank-sum test).

The hamster data set had less sequencing coverage per cell (34,181 mean reads; Table S2). Despite this limitation, viral transcripts were found. As in the mouse data set, *Orf10* was the most abundant viral transcript detected (1,066 *Orf10^+^* cells) and was used to define viral^+^ populations (Table S3). Ciliated epithelial cells (smallest cluster, 27 cells) had the highest percentage of *Orf10* ^+^ cells (29.6%, *n* = 8) (Fig. S2A) followed by *Marco* ^+^ alveolar cells (15.8%, Fig. 1F). These proportions are lower than those observed in K18-hACE2, where 55.3% of the ciliated cells were *Orf10^+^* (38 cells) (Fig. S1G). 8.3% of hamster *Sftpc* ^+^ alveolar type II cells contained *Orf10* (Fig. S2B). We found that 4.4% of the hamster B cells and 6.4% of the myocytes were positive for *Orf10* expression (Fig. S2C and D). 7.4% of *C1qb^+^* macrophages, 5.2% of *Cdh5* ^+^ endothelial cells, and 6.4% of fibroblasts were positive for *Orf10* (Fig. 1G and H; Fig. S2E). By comparing viral^+^ and viral^−^ cells within the *C1qb* ^+^ macrophage cluster, we identified the most DE host genes (35 genes), including *Cxcl10*, *Isg15*, *Ifit2*, and *Irf7* (Fig. 1G; Table S7). In *Marco^+^* alveolar cells, we found 19 host (e.g., *Cxcl10*, *Isg15, Tnfsf10*) and four viral sDEGs, *Orf10, Orf1ab, N,* and *S* (Fig. 1F; Table S8). In macrophage clusters, several chemokine genes (e.g., *Cxcl10, Ccl4/5/8*) were upregulated in viral^+^ (6.8%) cells. In macrophages, the viral^+^ cells had increased expression of *Cd38*, *Ibsp, and Isg1,* and both *Orf10* and *Orf1ab* (Fig. 2E). Finding the sDEG *Cxcl10* increased in the viral^+^ fraction in two macrophage populations raises the possibility of cell-intrinsic chemokine regulation in this data set. By comparing viral^+^ (*6.4*%) with viral^−^ myocytes, we found viral genes *Orf10, Orf1ab, Orf6, M, S,* and *N* were DE but no host genes were (Fig. S2D). In the *Sftpc* ^+^ alveolar type II cluster, we found several host sDEGs, including *Vim* and *Ghrl* and viral genes *Orf10* and *N* by comparing viral^+^ cells (*Orf10,* 8.3%) to viral^−^ cells (Fig. S2B). Notably, in the *Cd8^+^* cell cluster, we found no differences in host/viral expression between viral^+^ (4.4%) and viral^−^ cells (Fig. S2G).

### Human, AGM, ferret, and macaque BALF data sets

Next, we examined BALF cells from six human patients with severe/critical COVID-19 infection (12) (Table 1). Analysis of 51,555 cells (81,378 mean reads (host/viral, Table S2) detected six major clusters: *APOC1^+^* (58.2%), T cells (15.9%), B cells (12.3%), neutrophils (7.5%), *MUC5AC^+^* goblet cells (3.3%), and *FOXJ1^+^* cells (2.1%) (Fig. 2A). We found expression of viral RNA in all clusters. *FOXJ1^+^* cells (5.1%) and B cells (2.1%) contained the highest percentage of viral RNA^+^ cells, followed by neutrophils (1.8%), *MUC5AC^+^* goblet cells (1.7%), *APOC1^+^* macrophages (1.1%), and T cells (0.8%) (Fig. 2B through D; Fig. S3A through C). We found that 1.3% of cells had detectable *N* gene expression (the most abundant viral transcript in this data set) (Table S3).

In the human data set, we detected significant differences in host gene expression between viral^+^ and viral^−^ populations in epithelial cells, *MUC5AC* ^+^ goblet cells (Fig. 2B) and *FOXJ1* ^+^ ciliated cells, independently (Fig. 2C). Goblet cells had 16 sDEGs including the host genes *APOE, CYP27A1, C1QA*, and the viral transcript *N* (Fig. 2B; Table S9). Ciliated cells had 15 sDEGs (Fig. 2C; Table S10) including host genes *DDIT3, SPP1, TNFRSF12A*, and viral *N*. When we compared viral^+^ (2.1%) and viral^−^ B cells, we found four viral sDEGs: *ORF10, ORF1AB, N,* and the host gene *IGLV3* (Fig. 2D). In the neutrophil cluster, we compared viral^+^ (1.8%) and viral^−^ cells and found that the only sDEG was *N* (Fig. S3A). Similarly, we did not see evidence of host sDEGs in *APOC1^+^* or T cells (Fig. S3B and C). In this data set, we did not observe significant evidence of viral RNA-intrinsic regulation of IL1B, IL18, or CXCL10 (not sDEGs, viral^+^ vs viral^−^).

In AGM BALF, we defined eight clusters: APOC1^+^ macrophages (38.9%), T cells (36.8%), B cells (11.8%), endothelial cells (5.3%), alveolar macrophages (3.8%), goblet cells (1.9%), fibroblasts (1.1%), and ciliated cells (0.3%) (Fig. S3D). In this data set, there was less sequencing coverage per cell (22,225 mean reads; Table S2). Despite this, we found viral transcripts across clusters, including APOC1^+^ macrophages (4.3%), T cells (0.7%), B cells (5.1%), endothelial cells (2.2%), alveolar macrophages (0.8%), goblet cells (0.7%), fibroblasts (0.6%), and ciliated cells (0.3%) (Fig. S3E through K). The most represented viral RNA was ORF10 (2.7%) (Table S3). The APOC1^+^ macrophage cluster had the most sDEGs (30 genes, viral^+^ versus viral^−^) with 27 host genes (e.g., CXCL10, ISG15, IRF7) (Fig. S3E) and three viral genes, *ORF10*, *ORF1AB*, and *N* (Fig. S3E; Table S11). Like our findings in the hamster data set, CXCL10 was a sDEG increased in AGM viral^+^ macrophage fractions, highlighting the possibility of a cell-intrinsic viral RNA effect in macrophages. In the alveolar macrophage cluster, we found five sDEGs, four host genes, and ORF10 were increased in viral^+^ cells (0.8%) (Fig. S3I).

In the goblet cell cluster, we found two host sDEGs, RARRES1 and COL1A2, but no difference in viral expression between viral^+^ and viral^−^ cells (Fig. S3F). In the B cell cluster, we found six sDEGs between viral^+^ and viral^−^ populations (three host, three viral) (Fig. S3G). In the endothelial cell cluster, we found two sDEGs, (LGALS3 and ORF10, Fig. S3H). Although we found evidence of viral RNA in the T cell cluster, there were no host sDEGs between viral^+^ and viral^−^ populations (Fig. S5J). Fibroblasts had only one significant DEG, ORF10 when we compared viral^+^ to viral^−^ cells (Fig. S5K). In the ciliated cell cluster, there were no sDEGs found.

Analysis of ferret (*Mustela putorius furo*) BALF data (36,133 cells) (7) (Table 1) revealed nine major cell types: *CD68* ^+^ macrophages (50.2%), T cells (21.2%), *SCGB1A1^+^* epithelial cells (10.8%), *S100A12* ^+^ macrophages (10.7%), erythroid cells (3.8%), neutrophils (1.2%), *EPCAM* ^+^ epithelial cells (1.1%), ciliated cells (0.2%), and fibroblasts (0.01%) (Fig. S4A). We found only three viral^+^ cells (*ORF1AB* and *N*) in the entire data set; these were constrained to the *CD68^+^* + *S100A12^+^* macrophage clusters (Fig. S4B). Despite examining the combined *CD68* ^+^ and *S100A12* ^+^ macrophage population, no sDEGs were found due to the low viral^+^ (0.01%) cell number (Fig. S4B).

Rhesus macaque BALF also showed low mean reads per cell (23,697) (Table S2). Downstream analysis of 10 clusters (Fig. S4C) showed only two viral transcripts (ORF10) in the data set, both in the MARCO^+^ macrophage cluster (Fig. S4D). In the MARCO^+^ macrophage cluster, only the viral transcript ORF10 was identified as a viral sDEG with no differences in host genes due to the low viral^+^ cell representations when compared viral^+^ (0.003%) versus viral^−^ (Fig. S4D).

To compare sDEG identities between animals and human data, we created a Venn diagram (Fig. S5). We assessed the epithelial cell cluster for each species due to the presence of human sDEGs between viral^+^ and viral^−^ populations in the human epithelial cell cluster. Some common genes can be observed between several species.

### PCA and line chart

**Fig 3:**
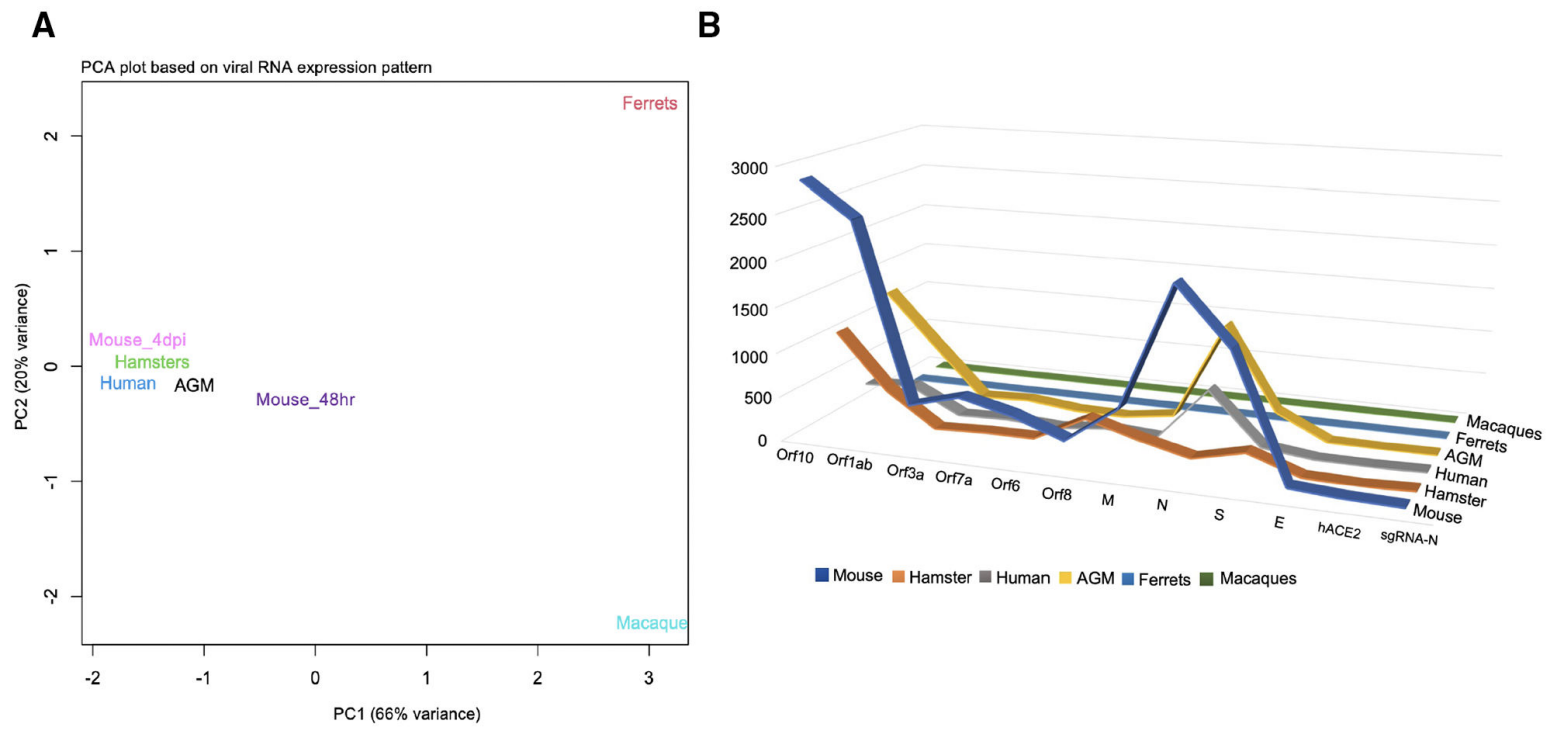
Human patients and animal models evaluation based on the distribution of viral RNA. (**A**) Principal component analysis (PCA) based on the gene expression patterns of 12 viral transcripts in the entire data set for humans and animals. Here, every point represents the total distribution of viral RNA for a species. The plot displays a percentage of the total variation in the animal models and human individuals. Where PC1 explains 68% of the total variance and PC2 explains 25%. Proximity to one another is indicative of shared variance. (**B**) A 3D line chart to visualize the best human COVID-19 recapitulating model based on the viral infection. Considered the 12*6 matrix here.

Based on the gene expression patterns of 12 viral transcripts in the infected whole lung and BALF data (Table S3), we conducted PCA (15) to identify the model that closely resembles human data. K18-hACE2 at both 4 dpi and 48 hours post infection, hamster, AGM, and human clustered together at the left, indicating close correlation (Fig. 3A). The ferret and macaque models are distant from the human model indicating they are a poor representation of human disease due to the low presence of viral RNA (Fig. 3A).

We also assembled a line chart to validate the best human COVID-19 model based on the quantity of each viral transcript (Table S3) in each data set (Fig. 3B). We found the K18-hACE2 model had more similar viral transcript distribution to human samples than any other model. These results further support the conclusion drawn from the PCA, that the K18-hACE2 model most closely approximates human infection.

### SARS-CoV-2 replication in infected samples

#### Human patients

**Fig 4:**
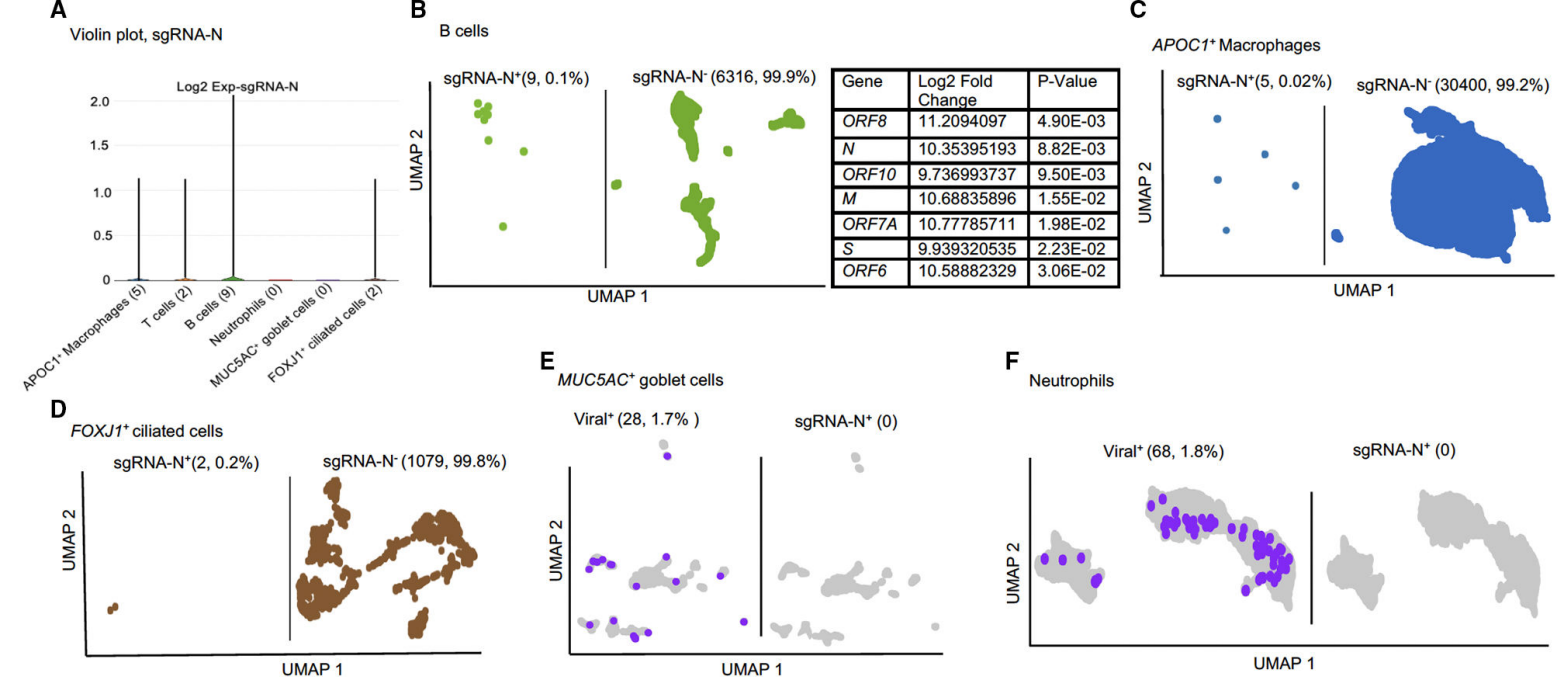
Analysis of the *sgRNA-N* expression in the infected human BALFs. (**A**) Violin plot showing the *sgRNA-N* expressing cell clusters in infected human patients BALF using single-cell RNA-seq. (**B–D**) UMAPs showing *sgRNA-N^+^* and *sgRNA-N* ^−^ cells and tables indicating significantly upregulated genes in *sgRNA-N* ^+^ populations compared with *sgRNA-N* ^-^ cells in the (**B**) B cell (**C**) *APOC1* ^+^ macrophage cell clusters. (**D and E**) UMAPs displaying the expression of viral^+^ and *sgRNA-N* ^+^ cells in the (**D**) *MUC5AC^+^* goblet cell and (**E**) neutrophil clusters.

The detection of *sgRNA* is a prerequisite for the functional investigation of viral replication (4, 15). Thus, *N*-gene subgenomic RNA (s*gRNA-N*) was used as an indication of viral replication (Fig. 4A). To investigate the distributions of *sgRNA-N*, we analyzed the sequence coverage of *sgRNA-N* across human patients and animal models (Fig. S6). We found consistent sequence coverage across patients and animals, which permits us to investigate the expression of *sgRNA-N* as a correlate to viral replication. *sgRNA-N* was detected in several human cell types, including B cell (nine cells, 0.1%), *APOC1^+^* (five cells, 0.02%), T cells (two cells, 0.02%), *FOXJ1^+^* cells (two cells, 0.2%) (Fig. 4A). To identify sDEGs, we set a threshold of *sgRNA-N*>5. In the B cell population, we found seven significant DE viral transcripts, for example, *ORF10, N*, and *S* when comparing *sgRNA-N* ^+^ versus *sgRNA-N^-^* cells (Fig. 4B). Interestingly, in the *APOC1^+^* macrophage population, we did not find any significant DE viral or host genes between *sgRNA-N^+^* and *sgRNA-N^-^* populations (Fig. 4C). This may be due to the low total number of *sgRNA-N* ^+^ cells in the data set. We also observed no *sgRNA-N* expression in *MUC5AC* ^+^ goblet cells or neutrophils (Fig. 4D and E). However, we found 28 viral^+^ *MUC5AC* ^+^ goblet cells and 68 viral^+^ (defined by *N* gene expression) neutrophils, suggesting viral RNA can be detected on/in *MUC5AC* ^+^ goblet cells and neutrophils, but we did not find any evidence of *sgRNA-N*, indicating no viral replication occurring in these cells.

#### Animal models

*sgRNA-N* was found to be expressed in several cell types in the infected mouse and hamster whole lungs and AGM BALFs (Fig. S7A through C). Interestingly, there is an absence of *sgRNA-N* in the ferret and macaque BALFs (Fig. S7D and E). Like in human patients, the analysis of DEGs (threshold was *sgRNA-N*>5) in mouse fibroblasts showed 41 host and viral sDEGs (Fig. S6F), whereas hamster *Marco^+^* alveolar cells showed 10 sDEGs (Fig. S6G). Within the mouse *C1qa^+^* macrophage population, comparison of *sgRNA-N^+^* and *sgRNA-N* ^−^ fractions yielded viral sDEGs but no host sDEGs (Fig. S7H). This was also found in mouse erythroid cells (Fig. S7I) and hamster myocytes (Fig. S7J). We did not perform DEG analysis on the AGM BALF data set because it lacked sufficient *sgRNA-N^+^* cells (e.g., *sgRNA-N* <5) (Fig. S7C).

*sgRNA-N* was absent in mouse ciliated cells, which may be due to low cell numbers, though 21 viral *Orf10^+^* cells were identified (Fig. S8A). In the hamster infected lung, we found no evidence of *sgRNA-N* in viral^+^ *C1qb* ^+^ macrophages (*Orf10*, 7.4%), *Cd8* ^+^ T cells (*Orf10,* 4.4%), or ciliated cells (*Orf10*, 29.6%) (Fig. S8B through D), this is likely due to insufficient sequencing depth. In the infected AGM BALF, we did not find evidence of sgRNA-N expression in the alveolar macrophages (ORF10, 3.8%), goblet cells (ORF10, 1.9%), fibroblasts (ORF10, 1.1%), or ciliated populations (ORF10, 0.3%) due to the low viral^+^ cell representation and low sequencing depth (Fig. S8E through H). These data suggest that viral uptake occurs in these cells but not viral replication, which further argues against the use of these models to recapitulate human disease.

### Analysis of proinflammatory cytokines (*IL1B*, *IL18*, and *CXCL10*) and most abundant viral RNA

#### Human patients

**Fig 5:**
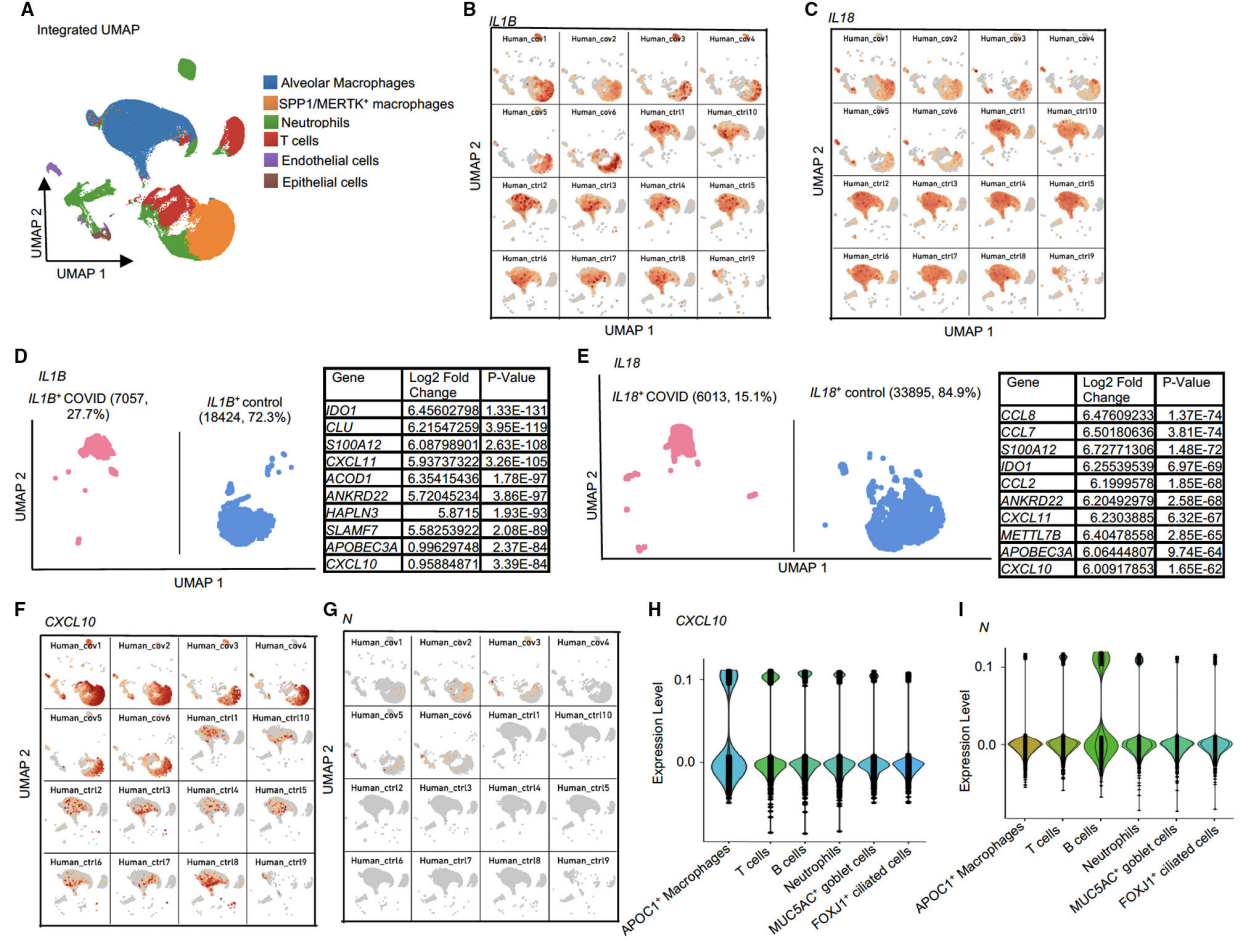
Analysis of several proinflammatory cytokines and the most abundant viral RNA *N* in the human BALFs. (**A**) UMAP showing the identified cell populations in integrated (COVID-19-infected and control) patient BALFs. (**B and C**) UMAPs for expression of (**B**) *CXCL10* and (**C**) *N* among the severe and healthy control samples. (**D and E**) Differential expression analysis of integrated BALFs within (**D**) *IL1B^+^* COVID-19-infected versus *IL1B^+^* control and (**E**) *IL18^+^* COVID-19-infected versus *IL18^+^* control. Tables enumerating the significantly upregulated genes found in the COVID-19-infected fractions. (**F and G**) Violin plots showing the expression of (**F**) host *CXCL10* and (**G**) viral *N* across different cell types in human BALF samples. Significancy is determined using a threshold adjusted *P* < 0.05 (*P*-value adjusted by multiple testing in the Wilcoxon rank-sum test).

Because comparing viral^+^ and viral^−^ cells did not reveal viral-intrinsic effects on *IL1B* or *IL18* expression, we examined proinflammatory cytokine expression across all clusters in the integrated (COVID-19-infected and control) human BALF data set (Fig. 5A). We observed that *IL1B* and *IL18* transcripts were largely confined to alveolar macrophages in the healthy control samples. In contrast, *IL1B* and *IL18* transcripts were expressed in the *SPP1/MERTK^+^* (inflammatory monocyte-derived) macrophage cluster in the COVID-19-infected samples (Fig. 5B and C). We compared *IL1B* ^+^ COVID-19 infected BALF (27.7%) and *IL1B* ^+^ healthy control BALF. We found host sDEGs but no differences in viral RNA (Fig. 5B; Table S12). Similarly, by comparing *IL18^+^* COVID-19-infected BALF (15.1%) with *IL18^+^* healthy controls, we found host sDEGs but no viral sDEGs (Fig. 5C; Table S13).

*CXCL10* is a biomarker of COVID-19 severity (16). Thus, we investigated *CXCL10* expression in the human integrated data set and found *CXCL10* was highly expressed in patients with severe/critical infections compared with healthy controls (Fig. 5F). We found *N* and *CXCL10* co-expressed in six clusters (Fig. 5G). The highest expression of *CXCL10* was in the *APOC1^+^* (Fig. 5H), whereas *N* expression was highest in the B cell cluster (Fig. 5I), indicating viral RNAs are not directly modulating *CXCL10* expression and suggesting they are linked by autocrine/paracrine interferon signaling.

#### Animal models

We also examined the expression of *IL1B* and *IL18* in mouse, hamster, AGM, ferret, and macaque integrated data sets (Fig. S9A through C, S4A and C). The analysis of mouse, hamster, and AGM data showed that *IL1B* and *lL18* were upregulated in the COVID-19-infected lungs and BALFs (Fig. S9D through I and S10A). Conversely, for macaque and ferret BALFs, we found discordant patterns of IL1B and IL18 expression (Fig. S10A through C). We compared *IL1B^+^*/*IL18* ^+^ cells in COVID-19-infected versus *lL1B^+^*/*IL18* ^+^ cells in healthy controls across models (Fig. S9D through I and Tables S14 through 20). We found host sDEGs (e.g., *CXCL2/10, IRF7*, *IFI27L2A*, *COL1A2, OAS1, ISG15/20*) across models and no viral sDEGs. Our data suggest viral RNA is not cell-intrinsically linked with *IL1B* or *IL18* expression in the infected lung or BALF.

*CXCL10* was highly expressed in the lungs and BALFs of only the infected animals, analogous to the human results (Fig. S10D through L). *CXCL10* and *ORF10* were expressed in different cells in each model. In the mouse model, *Cxcl10* expression was highest in fibroblasts (Fig. S11A), but *Orf10* was highest in alveolar macrophages (Fig. S11B). In hamster, *Cxcl10* expression was highest in myocytes (Fig. S11C), whereas *Orf10* expression was highest in ciliated cells (Fig. S11D). In AGM, *CXCL10* was most highly expressed in goblet cells (Fig. S11E), but *ORF10* was highest in endothelial cells (Fig. S11F). Due to low viral burden in ferret and macaque BALF, ORF10 expression was too low to draw conclusions on its impact on CXCL10 expression (Fig. S11G through J). The expression sites of *CXCL10* and the most abundant viral RNA (*N*/*ORF10*) are discordant in all models, suggesting autocrine/paracrine regulation. Importantly, *CXCL10* was a sDEG between viral^+^ and viral^−^ fractions in some subsets of macrophages in hamster, and AGM data also suggest cell-intrinsic viral RNA effects in these cell types.

## DISCUSSION

We performed an in-depth meta-analysis using a novel dual reference method of six published scRNA-seq data sets. We showed viral dissemination across various clusters in patient BALF and animal models. We found K18-hACE2 is the best approximation of severe human COVID-19, followed by hamster and AGM, showed viral replication in non-epithelial cell types, and described cells where viral uptake occurs and viral replication does not. We found no evidence of viral RNA being associated with *IL1B* or *IL18* mRNAs in infected lungs or BALFs and showed that viral RNAs do not explain *CXCL10* expression in infected patients. Thus, immune responses to viral RNA are likely driven by autocrine/paracrine signaling in some contexts.

We highlight significant viral transcriptomic differences in the BALF and lung tissue within several cell clusters and provide evidence of viral-intrinsic effects. We showed COVID-19 infection in K18-hACE2 and hamster whole lung in several non-epithelial tissues, including fibroblasts, endothelial cells, and macrophages. We found the previously described phenomena of aberrant macrophage and T cell responses and high chemokine expression in BALF samples from COVID-19 patients (12) could not be attributed to cell-intrinsic viral RNA effects. We analyzed viral^+^ cells that may or may not be due to infection. This may be due to residual ambient RNA or cell-associated viral RNA or viral RNA that is packaged in exosomes (15). Human samples were clinically defined as either moderate or severe/critical (12). Because our goal was to see how animal models mimic severe COVID-19, a limitation of the human data we do not know when the infection started. However, to attempt to control for viral burden, we compared the number of viral^+^ cells across the models (PCA plot). We found a smaller number of viral positive cells in the human macrophage clusters compared with the animal models, which may be due to sample collection methodology and tissue types available for human patients. Consistent with other single-cell RNA-seq studies, viral RNA has been found in multiple immune cells, including myeloid cells with phagocytic activity (neutrophil, monocyte, and macrophage) and lymphocytes without phagocytic activity (T, B, and NK cells) (17, 18). We also found viral genes in human epithelial cells, B cells, *APOC1^+^* macrophages, and T cells but did not find any evidence of host/viral sDEGs in either *APOC1^+^* macrophages or T cells. In AGMs, *APOC1^+^* macrophages were also infected, carrying the highest viral RNA burden. We did not find viral RNA in the BALF of ferrets or macaques, which is likely due to poor sequencing depth and limited viral infection/replication.

We demonstrated that SARS-CoV-2-infected K18-hACE2 had a higher percentage of viral RNA^+^ cells in all clusters compared to the infected hamster lung, AGM, ferret, and macaque BALF samples. Using PCA of ectopic viral RNA expression, we demonstrated that the K18-hACE2 model best approximated severe human COVID-19. Studies have also suggested that K18-hACE2 recapitulates the disease observed in COVID-19 patients (11, 13). In contrast, a recent *in vivo* study indicated that in K18-hACE2, Delta is the most pathogenic strain, followed by WA1, and Omicron is absent of clinical signs (4). Future studies are necessary to elucidate host-dependent pathogenicity and to examine changes in virulence over time in different variants of SARS-CoV-2. *sgRNA-N* was used to examine viral replication within each cell type. *sgRNA-N* was expressed in several clusters in the human and animal data. We found significant evidence of replication within human (B cells), mouse (fibroblasts), and hamster (*C1qa^+^* macrophages, erythroid cells, and myocytes) only. However, in the human data set, we observed that viral RNA was present, but we did not detect *sgRNA-N* in *MUC5AC* ^+^ goblet cells or neutrophils. Similarly, in animal models we found the presence of viral RNA in mouse ciliated cells and hamster *C1qb* ^+^ macrophages, *Cd8* ^+^ T cells, and ciliated cells but no replication. Moreover, we did not find evidence of viral replication in the AGM, ferret, or macaque data sets, consistent with minimal disease (7, 8).

Previous work has shown that *IL1B, IL18, and CXCL10* are induced by SARS-CoV-2 infection (19, 20) and contribute to COVID-19 pathogenesis (21, 22). In support of this, our analysis of data from mouse, hamster, AGM, and ferret models showed higher *IL1B* and *IL18* expression in infected BALF/lung when compared with uninfected controls. In human COVID-19, expression of *IL1B* and *IL18* shifted from mostly alveolar macrophages to *SPP1/MERTK^+^* macrophages. Unexpectedly low proinflammatory cytokine expression in the infected condition was found in the macaque model, this may be attributed to low sequencing depth and reduced viral mRNA. DEG analysis of *IL1B/IL18* ^+^ COVID-19-infected BALF/lung versus *IL1B/IL18* ^+^ control BALF/lung in the human, mouse, hamster, AGM, and ferret models showed host sDEGs and no viral sDEGs. No host or viral sDEGs were found for the macaque model. Moreover, viral RNAs are not linked to *IL1B* or *IL18* transcripts as they are found in different clusters. Our analysis also demonstrated that the most abundant viral transcripts (*N/ORF10*) are restricted to infected individuals. Also, *CXCL10* and *N/ORF10* were highly expressed in two distinct clusters in all infected lungs and BALFs, suggesting autocrine/paracrine *CXCL10* expression regulation. However, some of our data in the hamster and AGM models is suggestive of cell-intrinsic viral RNA effects on *CXCL10* expression, which may be due to these samples being obtained at earlier times of viral infection. It is highly likely that *CXCL10* expression is subject to both cell-intrinsic and -extrinsic regulatory mechanisms in the context of SARS-CoV-2 infection. We have employed a novel avenue of investigation that sheds light on the chemokine-storm phenomenon and the successful preclinical modeling of COVID-19 pathogenesis.

Collectively, our findings provide a high-resolution single-cell meta-analysis of several cell types in the SARS-CoV-2-infected lung and BALF. Despite the relatively low sequence depth for some samples, we identified previously unrecognized changes in gene expression and cellular interactions. We found that K18-hACE2 recapitulates severe COVID-19 in humans and can provide insight into disease pathogenesis. Results showed that viral RNAs are not linked to *IL1B* or *IL18* transcripts. We also demonstrated that viral RNAs do not explain *CXCL10* release alone, which may be regulated by autocrine/paracrine signaling. Expansion of the current atlas would be helpful to resolve functionally distinct cell subtypes with viral RNA and could reveal subtype-specific regions of the transcriptome that evade detection here.

## SUPPLEMENTAL MATERIAL

Supplemental material msphere.00375-23-s0001.pdf Supplemental text, Fig. S1 to S11, and Tables S1 to S3.

Supplemental tables msphere.00375-23-s0002.xlsx Tables S4 to S20.

ASM does not own the copyrights to Supplemental Material that may be linked to, or accessed through, an article. The authors have granted ASM a non-exclusive, world-wide license to publish the Supplemental Material files. Please contact the corresponding author directly for reuse.
