# Supplementary material for: Cell-intrinsic and -extrinsic effects of SARS-CoV-2 RNA on pathogenesis: single-cell meta-analysis
Source: mSphere. 2023 Sep 22;8(5):e00375-23. doi: 10.1128/msphere.00375-23 (PMC10597400; doi:10.1128/msphere.00375-23)
Supplement: Supplemental material — Supplemental text, Fig. S1 to S11, and Tables S1 to S3. [file msphere.00375-23-s0001.pdf]

Online Data Supplement

Cell-Intrinsic and -Extrinsic Effects of COVID-19 RNA on Pathogenesis: Single Cell Meta-Analysis

Mst Shamima Khatun[1], T. Parks Remcho[1], Xuebin Qin[2,3], and Jay K. Kolls[1]*

Methods

Dual custom references

The raw scRNA-seq data were processed using CellRanger version *V*6.1.2 (10X Genomics). The transcripts were aligned to a novel customized reference genome in which the *ORF10, ORF1AB, E, M, N, S, ORF3A, ORF6, ORF7A, ORF8, sgRNA-N*, and *hACE2* (MN985325.1, https://www.ncbi.nlm.nih.gov/nuccore/MN985325.1?report=genbank) were added as an additional gene to the human, mouse, hamster, AGM, ferrets, and macaques' reference genomes GRCh38, mm10-2020, MesAur1.0, ChlSab1.1, MusPutFur1.0, and mmul10, respectively. The 12 viral genes were appended to the human and five animal models annotation gtf files, and the genome was indexed using 'cellranger_mkref'.

Processing of scRNA-seq data

The Cell Ranger Single-Cell Software *V*6.1.2 (10x genomics) was used to perform downloaded raw fastq files. We aligned fastq files on customized reference file for each dataset with cellranger count. Cell Ranger output was directly analyzed with SoupX v1.6.2 with default parameters to remove contaminating ambient RNA. The ambient RNA corrected SoupX gene matrix outputs were then analyzed with Seurat v4.0.6 (https://github.com/ satijalab/seurat) R package. Barcodes were filtered that contained unexpectedly high counts of UMIs (Unique Molecular Identifiers) that were likely doublets. Low-quality cells were also removed to filter for noise from lung and BALF data. In each model, gene expression was normalized and scaled using the SCTransform algorithm. Last, the principal component analysis (PCA) and UMAP projections were calculated for the samples, and clusters of cells were identified. Each of model datasets were analyzed separately.

Marker detection and differential expression analysis

To identify marker genes, we used Seurat "FindAllMarkers" function based on the Wilcoxon rank-sum test to select genes in each cluster that were upregulated relative to the other clusters. We considered threshold >0.5 log fold change compared with the other clusters and a

Bonferroni-adjusted p value of <0.05. To perform DEGs analysis, Seurat ‘subset’ function were used to extract the certain population/cell types of clusters using the Wilcoxon rank-sum test.

Venn Diagram

‘Venn Diagram Maker’ (https://goodcalculators.com/venn-diagram-maker/) was used to generate symmetric Venn diagrams to compute common epithelial cell type sDEGs for human and three animal models. Venn diagram consists of multiple overlapping closed curves, each representing a species. The curves are overlapped in every possible way, showing all possible overlapping sDEGs between the species.

PCA analysis

PCA is an unsupervised machine learning technique. Besides using PCA as a data preparation technique, we also used it to visualize data. PCA was performed with the R package ‘Plotting PCA’ at default parameters based on the viral transcripts’ dissemination of each model. The autoplot function (generic function to explore the genomic data) of this package was used to plot the model object in R. PC1 and PC2 are evaluated for each model vector and plotted. The percentages of variation accounted by each PC1 and PC2 were displayed on the axes.

Line chart

R package ‘Plotly’ (https://plotly.com/r/3d-line-plots/) was used to generate the 3-D line chart by using the number of twelve viral transcripts for patient and animal datasets. Where vertical (value) axis indicate the column hight and horizontal (catagory) axis indicates the number of twelve viral transcripts. The depth axis indicates the six patient and animal models.

IGV analysis

Integrative Genomics Viewer (IGV) v2.12.3 was used to analyze the coverage profiles of human and animal single-cell RNA-sequence over the *sgRNA-N* gene. Cellranger output (.bam) file was inputted to mapped with customized genome reference file for each human BALF and animal models data.

Supplementary Figures

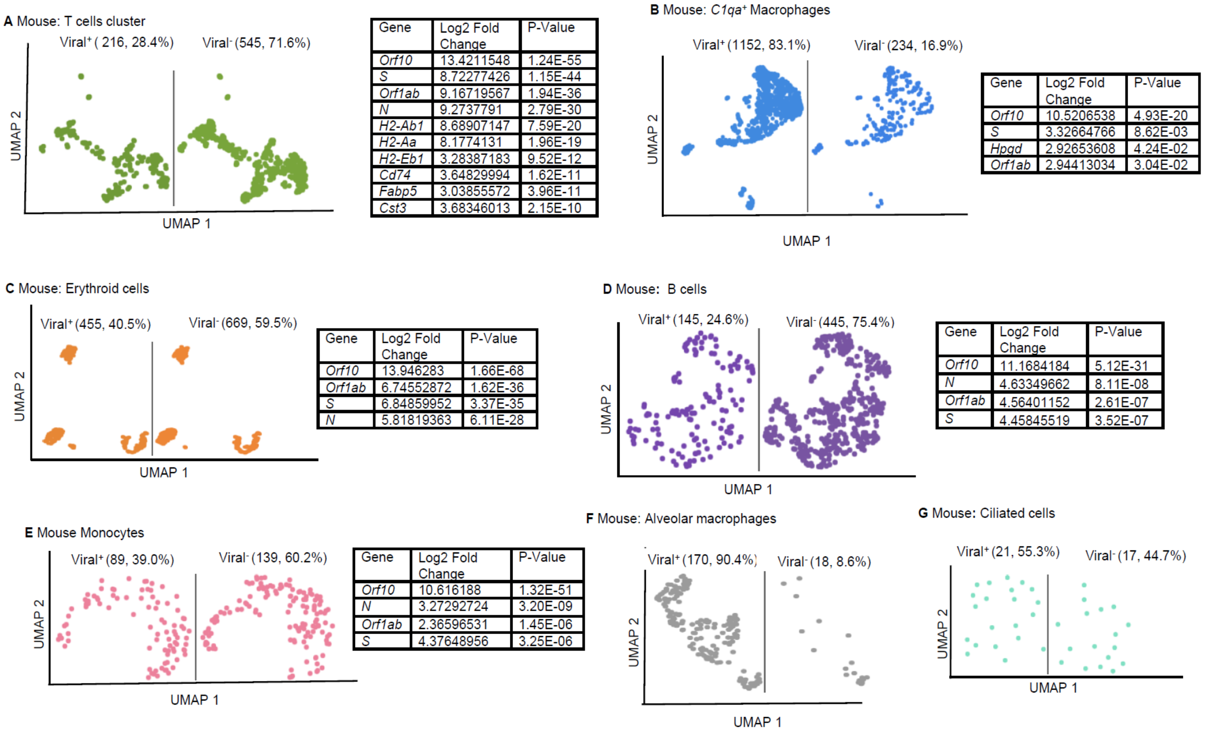

A

Gene	Log2 Fold Change	P-Value
Orf10	13.4211548	1.24E-55
S	8.72277426	1.15E-44
Orf1ab	9.16719567	1.94E-36
N	9.2737791	2.79E-30
H2-Ab1	8.68907147	7.59E-20
H2-Aa	8.1774131	1.96E-19
H2-Eb1	3.28387183	9.52E-12
Cd74	3.64829994	1.62E-11
Fabp5	3.03855572	3.96E-11
Cst3	3.68346013	2.15E-10

B

Gene	Log2 Fold Change	P-Value
Orf10	10.5206538	4.93E-20
S	3.32664766	8.62E-03
Hpgd	2.92653608	4.24E-02
Orf1ab	2.94413034	3.04E-02

C

Gene	Log2 Fold Change	P-Value
Orf10	13.946283	1.66E-68
Orf1ab	6.74552872	1.62E-36
S	6.84859952	3.37E-35
N	5.81819363	6.11E-28

D

Gene	Log2 Fold Change	P-Value
Orf10	11.1684184	5.12E-31
N	4.63349662	8.11E-08
Orf1ab	4.56401152	2.61E-07
S	4.45845519	3.52E-07

E

Gene	Log2 Fold Change	P-Value
Orf10	10.616188	1.32E-51
N	3.27292724	3.20E-09
Orf1ab	2.36596531	1.45E-06
S	4.37648956	3.25E-06

Figure S1. Differential expression analysis comparing viral$^+$ and viral$^-$ in various cell types from infected mouse lungs. (*A-G*) UMAP showing viral+ and – cells (*Orf10*) in the (*A*) T cell, (*B*) *C1qa*$^+$ macrophage, (*C*) erythroid cell, (*D*) B cell, (*E*) monocyte, (*F*) alveolar macrophage, and (*G*) ciliated cell clusters. The absence of gene tables for alveolar macrophages and ciliated cells indicates that we did not find significant genes following DEG analysis of these clusters. The table demonstrates significant genes from DEG analysis within the viral+ and – cells. The percentage indicated is the numeric fraction of viral$^{+/-}$ cells within the same cell type cluster.

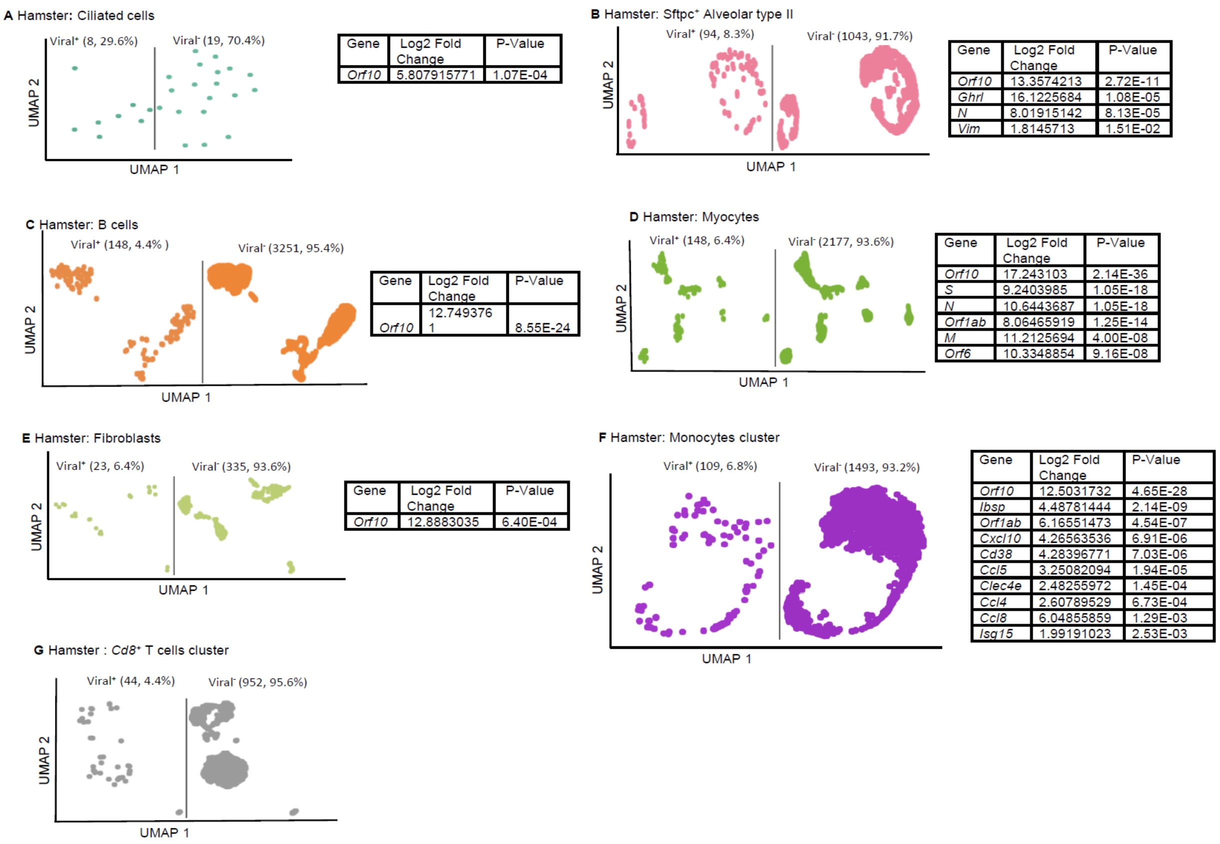

A

Gene	Log2 Fold Change	P-Value
Orf10	5.807915771	1.07E-04

B

Gene	Log2 Fold Change	P-Value
Orf10	13.3574213	2.72E-11
Ghrl	16.1225684	1.08E-05
N	8.01915142	8.13E-05
Vim	1.8145713	1.51E-02

C

Gene	Log2 Fold Change	P-Value
Orf10	12.749376 1	8.55E-24

D

Gene	Log2 Fold Change	P-Value
Orf10	17.243103	2.14E-36
S	9.2403985	1.05E-18
N	10.6443687	1.05E-18
Orf1ab	8.06465919	1.25E-14
M	11.2125694	4.00E-08
Orf6	10.3348854	9.16E-08

E

Gene	Log2 Fold Change	P-Value
Orf10	12.8883035	6.40E-04

F

Gene	Log2 Fold Change	P-Value
Orf10	12.5031732	4.65E-28
Ibsp	4.48781444	2.14E-09
Orf1ab	6.16551473	4.54E-07
Cxcl10	4.26563536	6.91E-06
Cd38	4.28396771	7.03E-06
Ccl5	3.25082094	1.94E-05
Clec4e	2.48255972	1.45E-04
Ccl4	2.60789529	6.73E-04
Ccl8	6.04855859	1.29E-03
Isg15	1.99191023	2.53E-03

Figure S2. Differential expression analysis comparing viral$^+$ and viral$^-$ across different cell types from, infected hamster lungs. (*A*-*G*) UMAP showing the viral+ and - cells (*Orf10*) in the (*A*) ciliated cells, (*B*) *Sftpc*$^+$ alveolar type II, (*C*) B cells, (*D*) myocytes, (*E*) fibroblasts, (*F*) monocytes, and (*G*) *Cd8*$^+$ T cells clusters. DEG analysis did not reveal any differences withing the *Cd8*$^+$ T cell cluster. The tables enumerate significant genes following DEG analysis within the viral+ and – cells. The percentage indicated is the numeric fraction of viral$^{+/-}$ cells within the same cell type cluster.

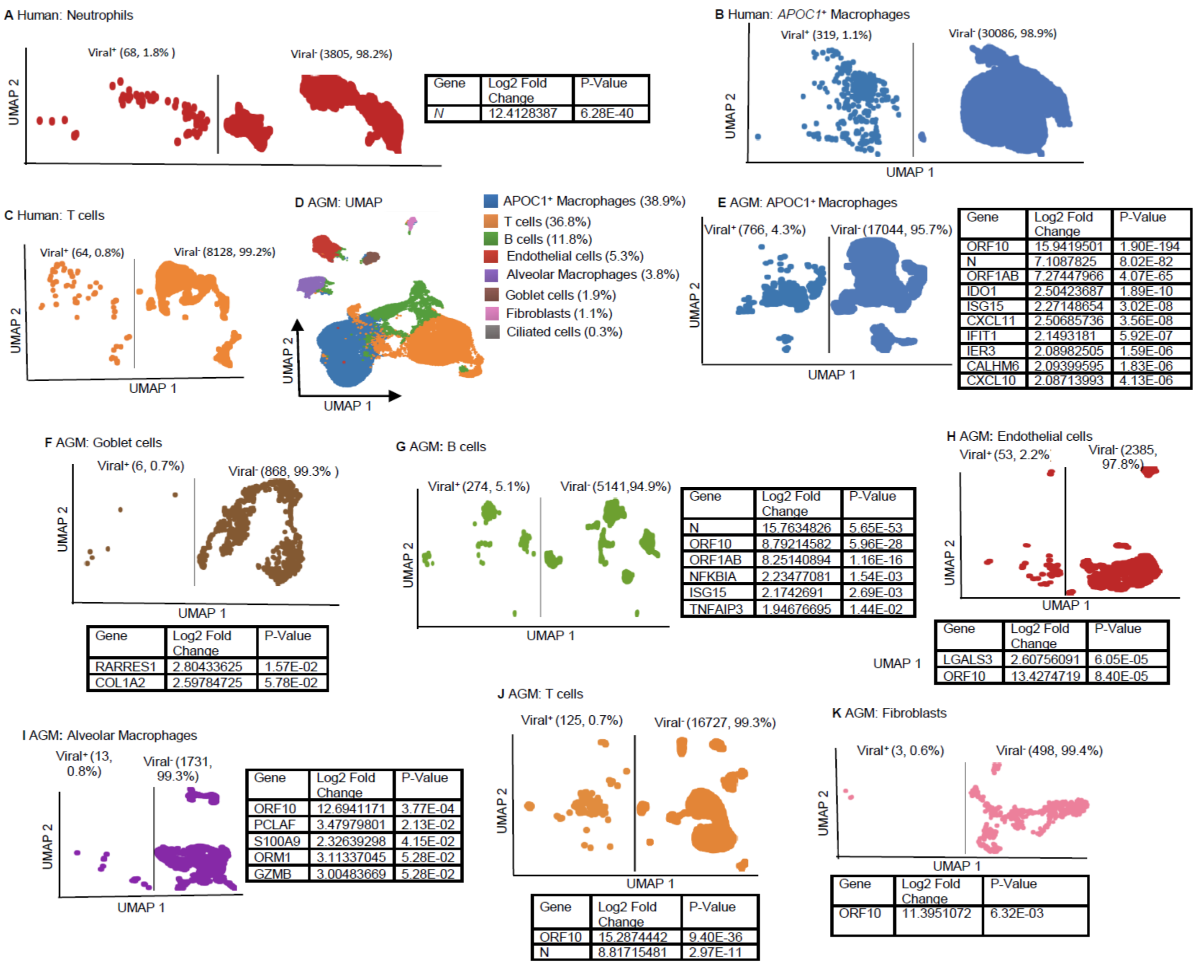

(A)

Gene	Log2 Fold Change	P-Value
N	12.4128387	6.28E-40

(E)

Gene	Log2 Fold Change	P-Value
ORF10	15.9419501	1.90E-194
N	7.1087825	8.02E-82
ORF1AB	7.27447966	4.07E-65
IDO1	2.50423687	1.89E-10
ISG15	2.27148654	3.02E-08
CXCL11	2.50685736	3.56E-08
IFIT1	2.1493181	5.92E-07
IER3	2.08982505	1.59E-06
CALHM6	2.09399595	1.83E-06
CXCL10	2.08713993	4.13E-06

(F)

Gene	Log2 Fold Change	P-Value
RARRES1	2.80433625	1.57E-02
COL1A2	2.59784725	5.78E-02

(G)

Gene	Log2 Fold Change	P-Value
N	15.7634826	5.65E-53
ORF10	8.79214582	5.96E-28
ORF1AB	8.25140894	1.16E-16
NFKBIA	2.23477081	1.54E-03
ISG15	2.1742691	2.69E-03
TNFAIP3	1.94676695	1.44E-02

(H)

Gene	Log2 Fold Change	P-Value
LGALS3	2.60756091	6.05E-05
ORF10	13.4274719	8.40E-05

(I)

Gene	Log2 Fold Change	P-Value
ORF10	12.6941171	3.77E-04
PCLAF	3.47979801	2.13E-02
S100A9	2.32639298	4.15E-02
ORM1	3.11337045	5.28E-02
GZMB	3.00483669	5.28E-02

(J)

Gene	Log2 Fold Change	P-Value
ORF10	15.2874442	9.40E-36
N	8.81715481	2.97E-11

(K)

Gene	Log2 Fold Change	P-Value
ORF10	11.3951072	6.32E-03

Figure S3. Differential expression analysis comparing viral$^+$ and viral$^-$ cells of different types derived from human patients with severe COVID-19 and infected AGM BALFs. (*A-C*) UMAP showing the viral+ and - cells (Human: *N*, AGM: *Orf10*) in (*A*) human neutrophils, (*B*) human *APOC1*$^+$ macrophages, and (*C*) human T cells clusters. (*D*) UMAP plot displayed the major cell types in eight clusters for AGM BALF samples, (*E-K*) UMAP shown the viral+ and - cells in the (*E*) AGM *APOC1*$^+$ macrophages, (*F*) AGM goblet cells II, (*G*) AGM B cells, (*H*) AGM endothelial cells, (*I*) AGM alveolar macrophages, (*J*) AGM T cells, and (*K*) AGM fibroblasts. The tables demonstrate significant genes from DEG analysis within the viral+ and – cells. The percentage indicated is the numeric fraction of viral$^{+/-}$ cells within the same cell type cluster.

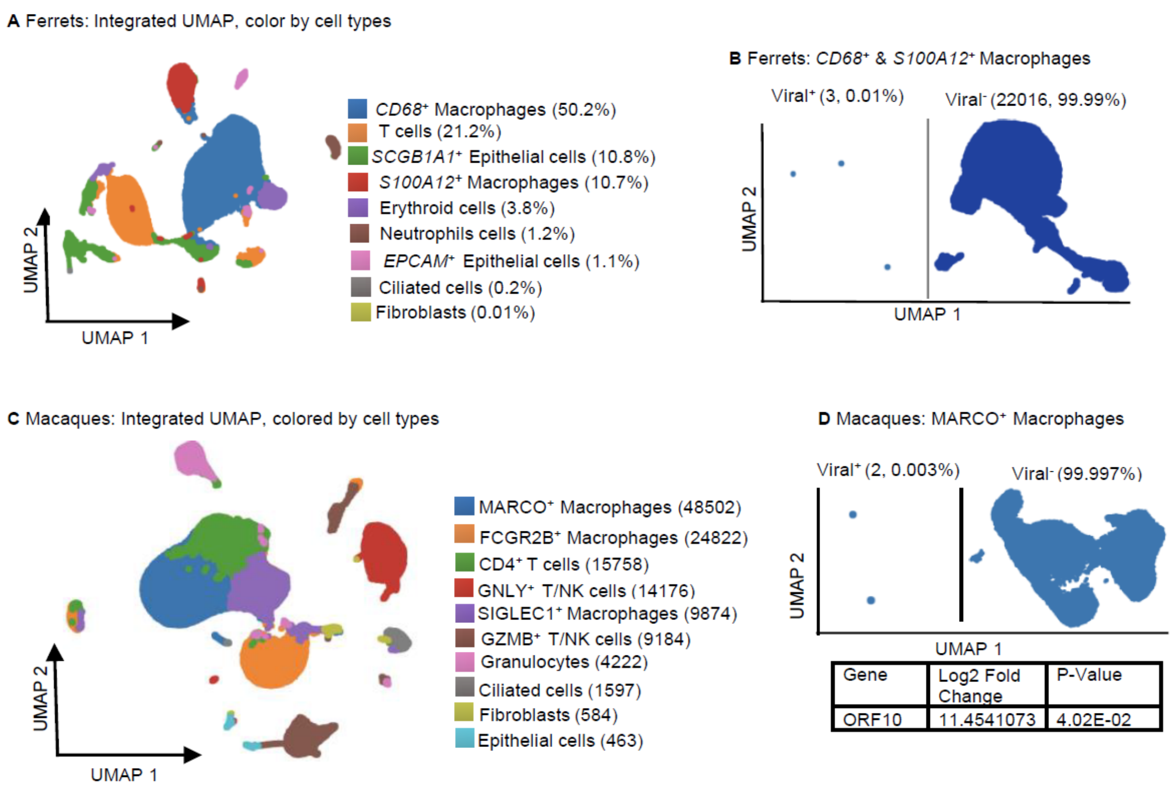

Figure S4. DEG analysis of ferret and macaque BALF cells. (*A*) UMAP plot of identified cell populations in ferret integrated (COVID & control) BALF samples. Colors represent individual cell types and are described in the legend. (*B*) UMAP showing the viral+ and - cells in the ferrets *CD68*$^+$ & *S100A12*$^+$ macrophage cluster with no significant DEGs. (*C*) UMAP plot of identified cell clusters in macaque integrated (COVID & control) BALF samples. Ten individual cell types were profiled in this dataset. (*D*) UMAP shown the viral+ and - cells in the macaques MARCO$^+$ macrophage cluster. Tables show the viral gene ORF10 Is DE however, no host genes are DE between these groups.

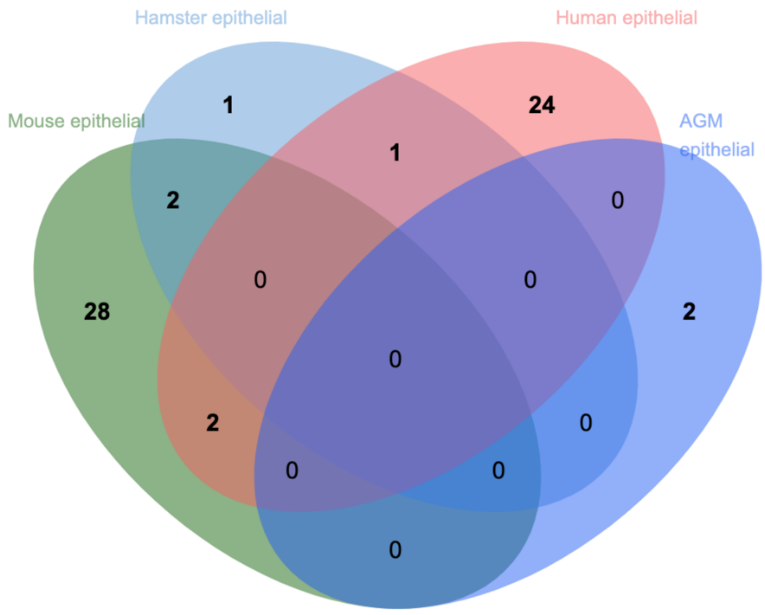

Figure S5. Venn diagram comparing epithelial cell type sDEGs for human and three animal models. Numbers represent the sDEGs between viral^{+} and viral^{-} epithelial cells for each species. Human gene nomenclature was used for generating the diagram. Ferret and Rheses macaque data were excluded due to minimal viral loads.

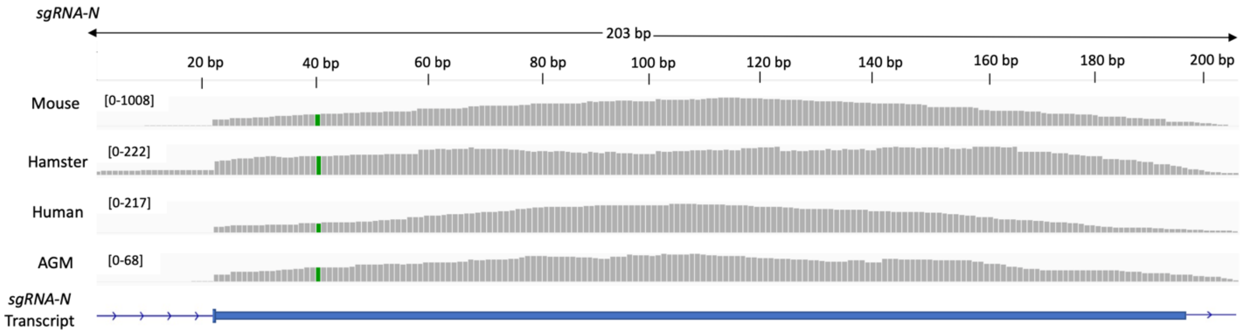

Figure S6. Coverage profiles of human and animals single-cell RNA-sequence over the *sgRNA-N* gene (203 base pairs (bp)). The annotation shown for the 3'-UTR elongated by 20 bp.

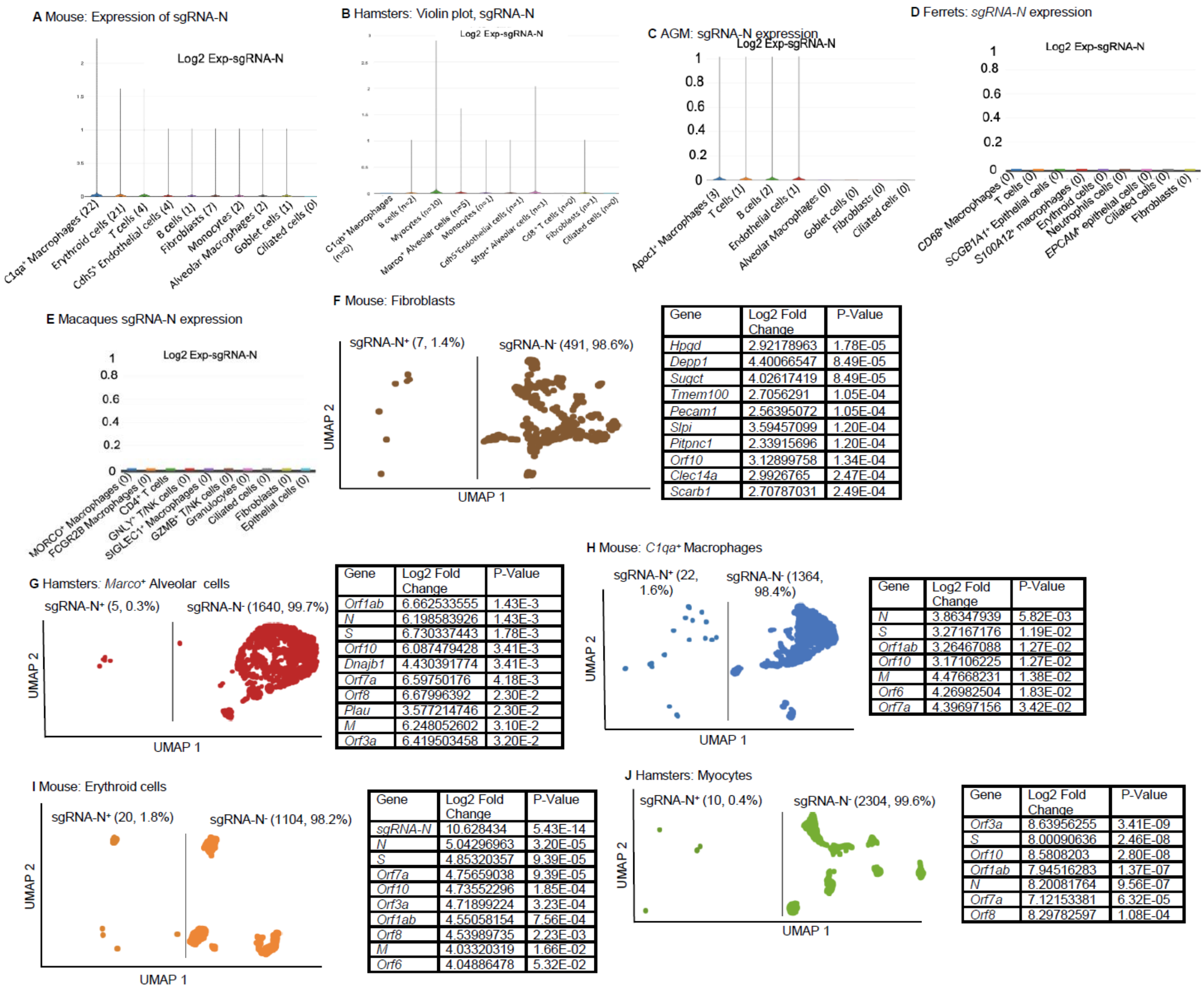

F Mouse: Fibroblasts

Gene	Log2 Fold Change	P-Value
Hpgd	2.92178963	1.78E-05
Depp1	4.40066547	8.49E-05
Sugct	4.02617419	8.49E-05
Tmem100	2.7056291	1.05E-04
Pecam1	2.56395072	1.05E-04
Slpi	3.59457099	1.20E-04
Pitpnc1	2.33915696	1.20E-04
Orf10	3.12899758	1.34E-04
Clec14a	2.9926765	2.47E-04
Scarb1	2.70787031	2.49E-04

G Hamsters: *Marco*+ Alveolar cells

Gene	Log2 Fold Change	P-Value
Orf1ab	6.662533555	1.43E-3
N	6.198583926	1.43E-3
S	6.730337443	1.78E-3
Orf10	6.087479428	3.41E-3
Dnajb1	4.430391774	3.41E-3
Orf7a	6.59750176	4.18E-3
Orf8	6.67996392	2.30E-2
Plau	3.577214746	2.30E-2
M	6.248052602	3.10E-2
Orf3a	6.419503458	3.20E-2

H Mouse: *C1qa*+ Macrophages

Gene	Log2 Fold Change	P-Value
N	3.86347939	5.82E-03
S	3.27167176	1.19E-02
Orf1ab	3.26467088	1.27E-02
Orf10	3.17106225	1.27E-02
M	4.47668231	1.38E-02
Orf6	4.26982504	1.83E-02
Orf7a	4.39697156	3.42E-02

I Mouse: Erythroid cells

Gene	Log2 Fold Change	P-Value
sgRNA-N	10.628434	5.43E-14
N	5.04296963	3.20E-05
S	4.85320357	9.39E-05
Orf7a	4.75659038	9.39E-05
Orf10	4.73552296	1.85E-04
Orf3a	4.71899224	3.23E-04
Orf1ab	4.55058154	7.56E-04
Orf8	4.53989735	2.23E-03
M	4.03320319	1.66E-02
Orf6	4.04886478	5.32E-02

J Hamsters: Myocytes

Gene	Log2 Fold Change	P-Value
Orf3a	8.63956255	3.41E-09
S	8.00090636	2.46E-08
Orf10	8.5808203	2.80E-08
Orf1ab	7.94516283	1.37E-07
N	8.20081764	9.56E-07
Orf7a	7.12153381	6.32E-05
Orf8	8.29782597	1.08E-04

Figure S7. Analysis of the *sgRNA-N* expression in the infected animal's lung and BALF. (*A-E*) violin plots for the expression in (*A*) mouse lung, (*B*) hamster lung, (*C*) AGM BALF, (*D*) ferret, and (*E*) macaque BALF. The plots represent the expression of viral *sgRNA-N* across different cell types found within the infected data. (*F-J*) UMAPs showing differentially expressed genes between *sgRNA-N*+ and – cells in the (*F*) mouse fibroblasts, (*G*) hamster *Marco*+ alveolar macrophages, (*H*) mouse *C1qa*+ macrophages, (*I*) mouse erythroid cells, and (*J*) hamster myocyte cells. The tables provide the significant genes upregulated in the *sgRNA-N*+ fraction. A gene is considered significant if it exhibits an adjusted P < 0.05 (P-value adjusted by multiple testing in the Wilcoxon rank-sum test). *sgRNA-N* = subgenomic RNA.

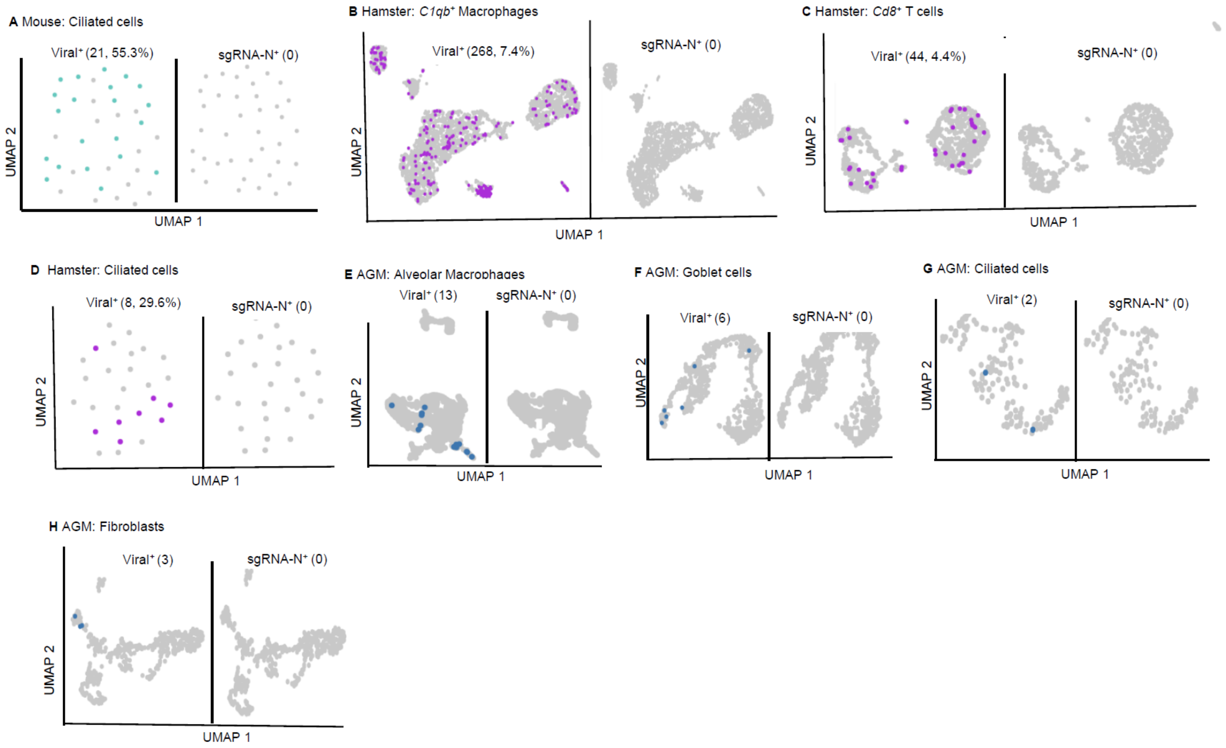

Figure S8. Examining the absence of *sgRNA-N* across different cell clusters described within infected animal lung and BALF samples. (*A-H*) UMAPs showing the expression of viral^{+} and *sgRNA-N*$^{+}$ cells in the animal data (*A*) mouse ciliated cells, (*B)* hamster *C1qb*$^{+}$ macrophages, (*C*) hamster *Cd8*$^{+}$ T cells, (*D*) hamster ciliated cells, (*E*) AGM alveolar macrophages, (*F*) AGM goblet cells, (*G*) AGM ciliated cells, and (*H*) AGM fibroblasts. A gene is considered significant if it has an adjusted P < 0.05 (P-value adjusted by multiple testing in the Wilcoxon rank-sum test).

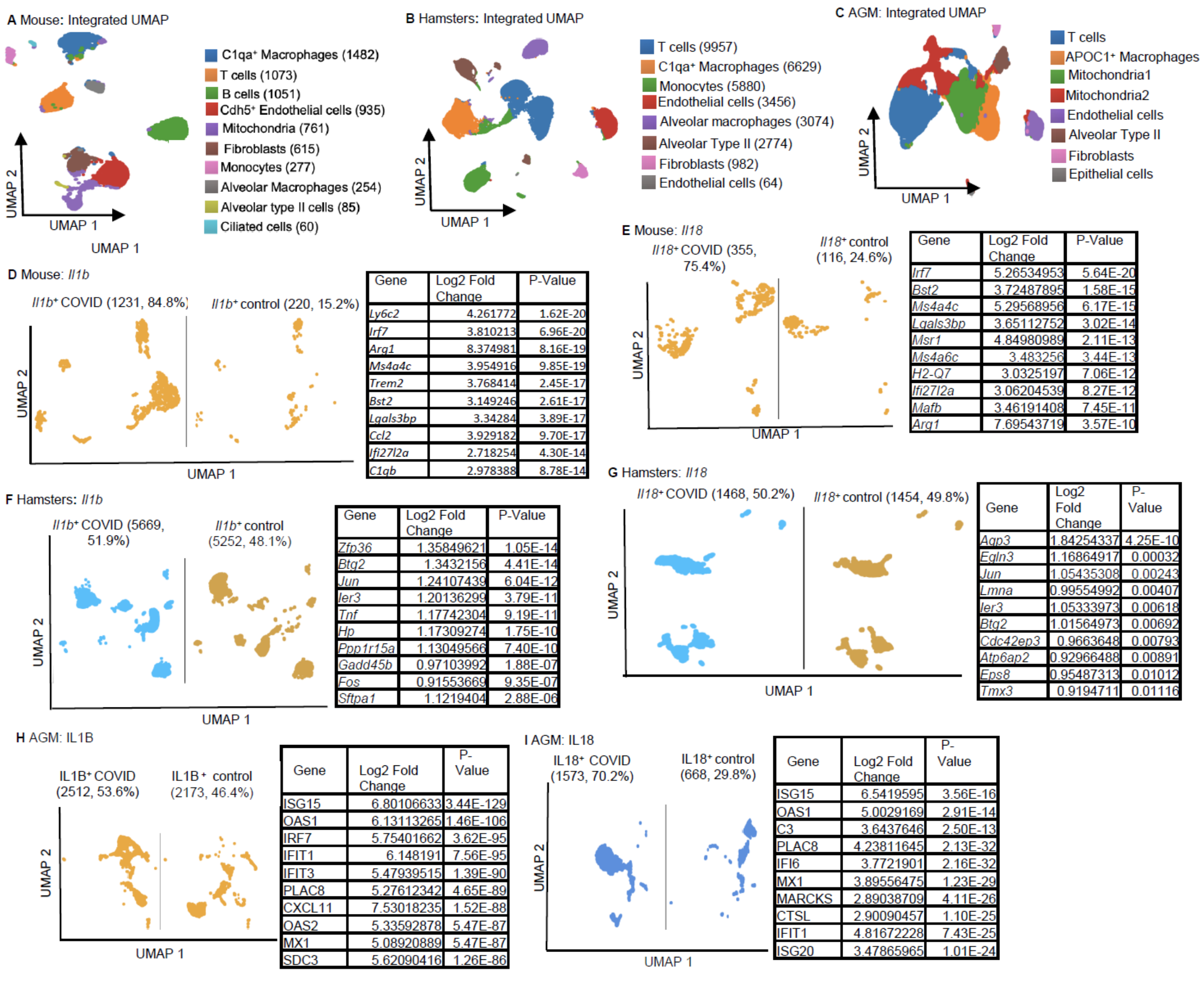

D

Gene	Log2 Fold Change	P-Value
Ly6c2	4.261772	1.62E-20
Irf7	3.810213	6.96E-20
Arg1	8.374981	8.16E-19
Ms4a4c	3.954916	9.85E-19
Trem2	3.768414	2.45E-17
Bst2	3.149246	2.61E-17
Lgals3bp	3.34284	3.89E-17
Ccl2	3.929182	9.70E-17
Ifi27l2a	2.718254	4.30E-14
C1qb	2.978388	8.78E-14

E

Gene	Log2 Fold Change	P-Value
Irf7	5.26534953	5.64E-20
Bst2	3.72487895	1.58E-15
Ms4a4c	5.29568956	6.17E-15
Lgals3bp	3.65112752	3.02E-14
Msr1	4.84980989	2.11E-13
Ms4a6c	3.483256	3.44E-13
H2-Q7	3.0325197	7.06E-12
Ifi27l2a	3.06204539	8.27E-12
Mafb	3.46191408	7.45E-11
Arg1	7.69543719	3.57E-10

F

Gene	Log2 Fold Change	P-Value
Zfp36	1.35849621	1.05E-14
Btg2	1.3432156	4.41E-14
Jun	1.24107439	6.04E-12
Ier3	1.20136299	3.79E-11
Tnf	1.17742304	9.19E-11
Hp	1.17309274	1.75E-10
Ppp1r15a	1.13049566	7.40E-10
Gadd45b	0.97103992	1.88E-07
Fos	0.91553669	9.35E-07
Sftpa1	1.1219404	2.88E-06

G

Gene	Log2 Fold Change	P-Value
Aqp3	1.84254337	4.25E-10
Egln3	1.16864917	0.00032
Jun	1.05435308	0.00243
Lmna	0.99554992	0.00407
Ier3	1.05333973	0.00618
Btg2	1.01564973	0.00692
Cdc42ep3	0.9663648	0.00793
Atp6ap2	0.92966488	0.00891
Eps8	0.95487313	0.01012
Tmx3	0.9194711	0.01116

H

Gene	Log2 Fold Change	P-Value
ISG15	6.80106633	3.44E-129
OAS1	6.13113265	1.46E-106
IRF7	5.75401662	3.62E-95
IFIT1	6.148191	7.56E-95
IFIT3	5.47939515	1.39E-90
PLAC8	5.27612342	4.65E-89
CXCL11	7.53018235	1.52E-88
OAS2	5.33592878	5.47E-87
MX1	5.08920889	5.47E-87
SDC3	5.62090416	1.26E-86

I

Gene	Log2 Fold Change	P-Value
ISG15	6.5419595	3.56E-16
OAS1	5.0029169	2.91E-14
C3	3.6437646	2.50E-13
PLAC8	4.23811645	2.13E-32
IFI6	3.7721901	2.16E-32
MX1	3.89556475	1.23E-29
MARCKS	2.89038709	4.11E-26
CTSL	2.90090457	1.10E-25
IFIT1	4.81672228	7.43E-25
ISG20	3.47865965	1.01E-24

Figure S9. Analysis of proinflammatory cytokines and the most abundant viral RNA, *Orf10,* across animal models. (*A*-*C*) UMAP plots displaying the major cell types found in the integrated (infected and control) datasets from (*A*) mouse, (*B*) hamster, and (*C*) AGM samples. Colors represent individual cell types and are depicted in legend. (*D*-*I*) UMAPs showing the percentage of *Il1b*⁺ and *Il18*⁺ cells in the COVID-infected and control data. Tables showing the upregulated genes in the *Il1b*⁺ and *Il18*⁺ COVID-infected fraction for each animal model (*D* and *E*) mouse, (*F* and *G*) hamster, and (*H* and *I*) AGM. A gene is considered significant if it achieves an adjusted P < 0.05 (P-value adjusted by multiple testing in the Wilcoxon rank-sum test).

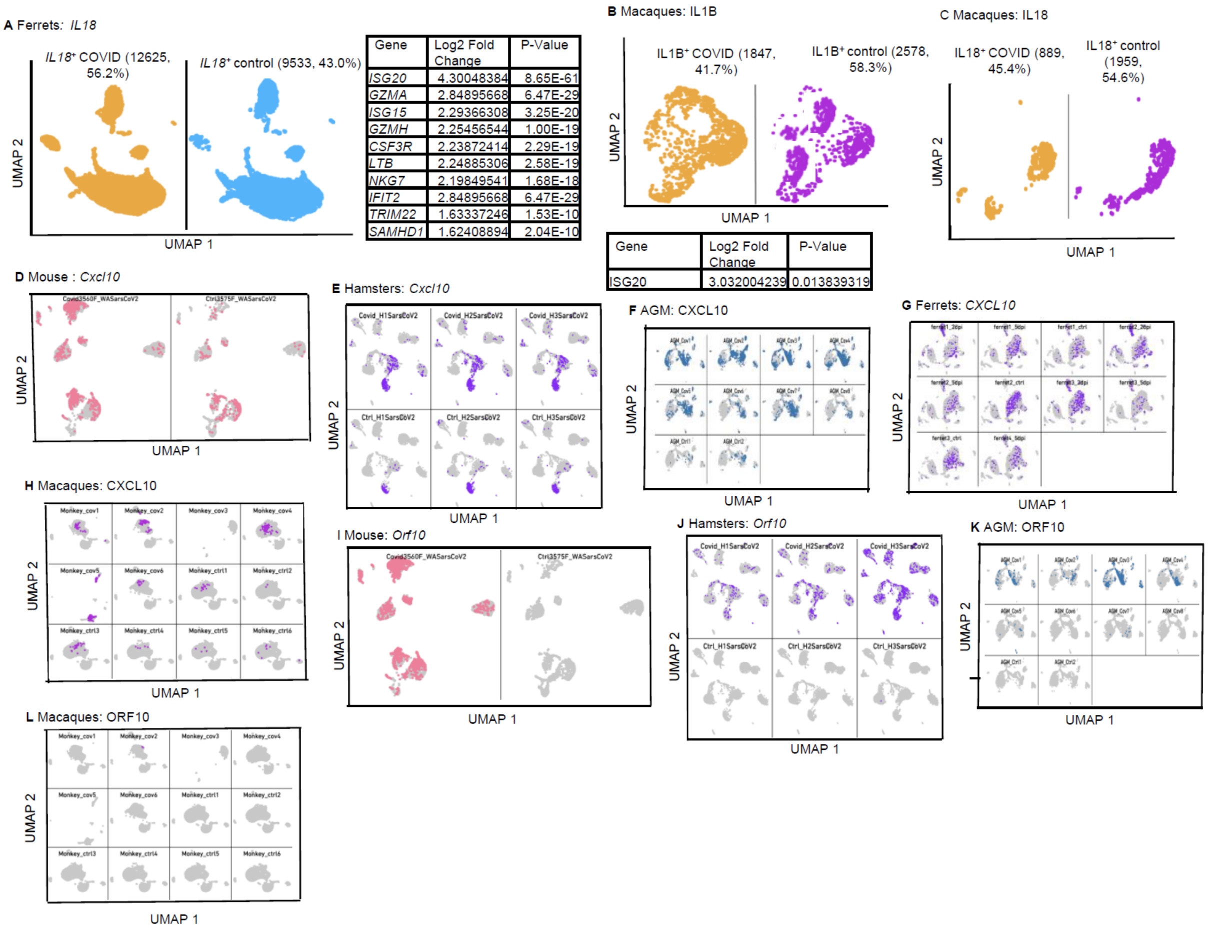

Gene	Log2 Fold Change	P-Value
ISG20	4.30048384	8.65E-61
GZMA	2.84895668	6.47E-29
ISG15	2.29366308	3.25E-20
GZMH	2.25456544	1.00E-19
CSF3R	2.23872414	2.29E-19
LTB	2.24885306	2.58E-19
NKG7	2.19849541	1.68E-18
IFIT2	2.84895668	6.47E-29
TRIM22	1.63337246	1.53E-10
SAMHD1	1.62408894	2.04E-10

Gene	Log2 Fold Change	P-Value
ISG20	3.032004239	0.013839319

Figure S10. Expression of *Il1b, Il18*, and *Cxcl10* and the most abundant viral RNA (*Orf10*) in the animal datasets. (*A*-*C*) UMAPs showing the percentage of *Il1b*$^{+}$ and *Il18*$^{+}$ cells in the COVID-infected and control data. Tables enumerating upregulated genes in the *Il1b*$^{+}$ or *Il18*$^{+}$ COVID-infected fraction for each model system: (*A*) ferret, (*B* and *C*) macaque. (*D*-*H*) UMAP demonstrating the expression of *Cxcl10* in the (*D*) mouse, (*E*) hamster, (*F*) AGM, (*G*) ferret, and (*H*) macaque derived samples. The different panels indicated *Cxcl10* expression in the individual sample for the infected and control data. (*I*-*L*) UMAPs showing the expression of the most abundant viral RNA, *Orf10,* in the (*I*) mouse, (*J*) hamster, (*K*) AGM, and (*L*) macaque data. Different panels are provided for infected and control data.

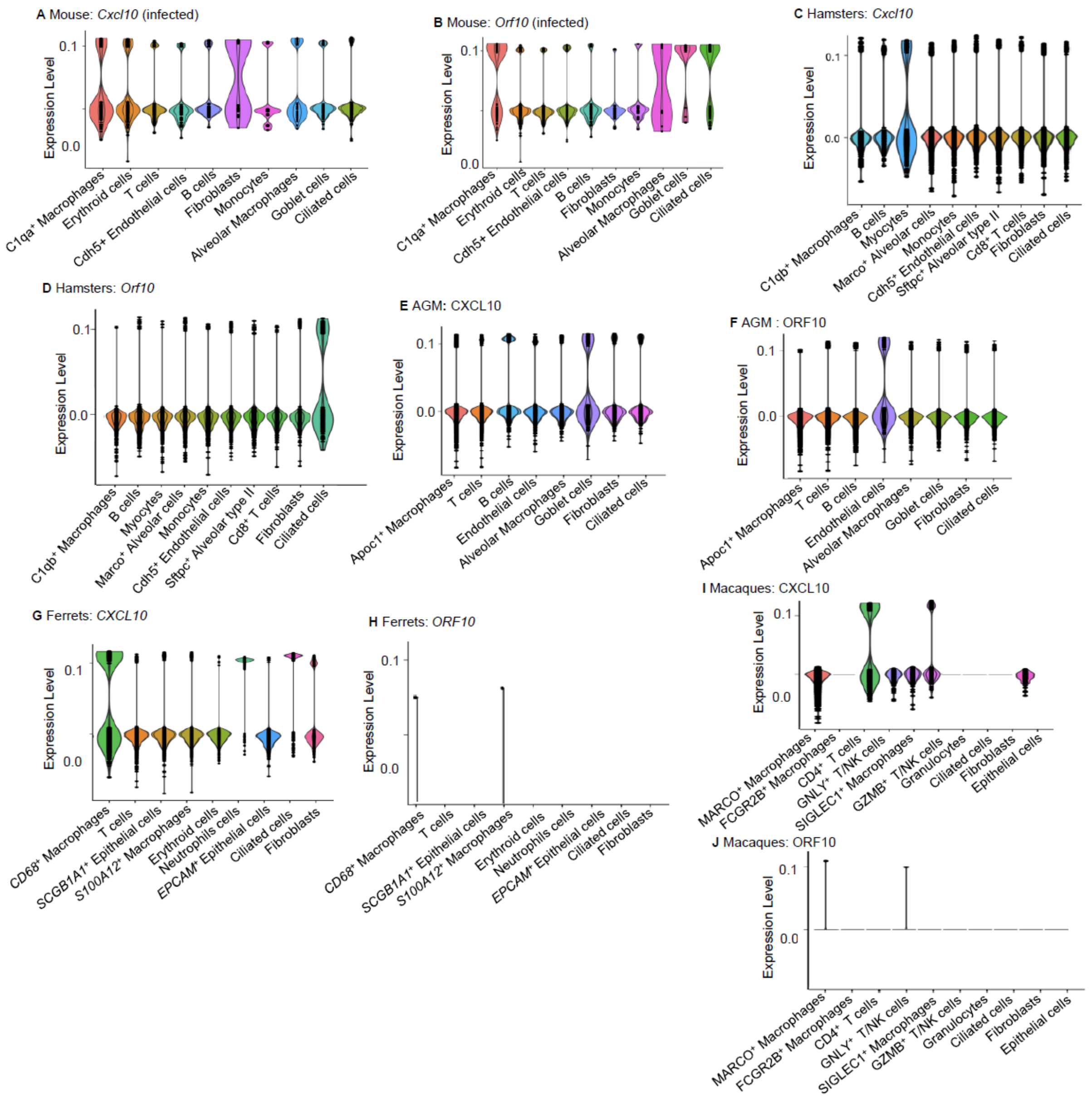

Figure S11. Expression of *Cxcl10* and most abundant viral RNA (*Orf10*) are discordant. (*A-J*) Violin plots showing expression levels of *Cxcl10* and *Orf10* in different cell type (shown as different clusters) in the infected (*A* and *B*) mouse whole lung, (*C* and *D*) hamster whole lung, (*E* and *F*) AGM BALF, (*G* and *H*) ferret BALF, and (*I* and *J*) macaque BALF. Colors representing individual cell types are described in the legend.

Supplementary Tables

Table S1: Clinical data information of the enrolled COVID-19 severe/critical patients

Parameters	S1	S2	S3	S4	S5	S6
Severity	Severity	Critical	Critical	Critical	Critical	Critical
Gender	Male	Male	Male	Female	Female	Male
Age	62	66	63	65	57	46
Symptom onset date	2020-1-11	2020-1-3	2020-1-8	2020-1-4	2020-1-21	020-1-21
First symptom	Fever/Cough	Fever/Cough	Fever/Cough	Fever/Cough / Chest distress	Dizziness/ Fever/ Chest distress	Fever/Cough
BALF sampling time	2020-1-22	2020-1-21	2020-1-22	2020-1-29	2020-1-29	2020-2-2
Outcome/Date	Cured (2020-2-27)	Death (2020-2-16)	Death (2020-2-16)	Cured (2020-3-9)	Cured (2020-3-7)	Cured (2020-3-8)
Chronic basic disease	None	Hypertension	Sleep apnea	Diabetes	None	None

Table S2. Total number of cells and reads for human individuals and 5 animal models

Models name	Cell types	#Total cells	#Reads
Human	Macrophages	30405	**1.** Number of Cells: **51,555 (6 samples)**<br>**2.** Total Number of Reads: **4,195,440,370**<br>**3.** Mean Reads per Cell: **81,378**<br>**Tec: BGISEQ-500**
	T cells	8192	
	B cells	6325	
	Neutrophils	3873	
	Muc5ac$^+$ cells	1679	
	Foxj1$^+$ cells	1081	
Hamsters	Macrophages	3627	1. Number of Cells: **16,382 (3 samples)**<br>2. Total Number of Reads: **559,958,478**<br>3. Mean Reads per Cell: **34,181**<br>**Tec: Illumina NovaSeq 6000**
	B cells	3399	
	Muscle cells	2314	
	Epithelial	1645	
	Monocytes	1602	
	Cdh5$^+$ EC	1277	
	Sftpc$^+$ Alveolar	1137	
	CD8$^+$ T cells	996	
	Fibroblasts	358	
	Ciliated cells	27	
Mouse	C1qa$^+$ Macrophages	1386	**1.** Number of Cells: **5,696 (1 sample)**<br>**2.** Total Number of Reads: **463,251,447**<br>**3.** Mean Reads per Cell: **81,329**<br>**Tec: Illumina NextSeq 2000**
	Erythroid cells	1124	
	T cells	761	
	Cdh5$^+$ EC	630	
	B cells	590	
	Fibroblasts	498	
	Monocytes	228	
	Alveolar Macrophages	188	
	Goblet cells	65	

	Ciliated cells	38	
AGM	$APOC1^+$ Macrophages	17810	**1.** Number of Cells: **45,779 (6 samples)**<br>**2.** Total Number of Reads: **1,017,457,350**<br>**3.** Mean Reads per Cell: **22,225**<br>**Tec: NextSeq 550**
	T cells	16852	
	B cells	5415	
	Endothelial cells	2438	
	Alveolar Macrophages	1744	
	Goblet cells	874	
	Fibroblasts	501	
	Ciliated cells	145	
Ferrets	$CD68^+$ Macrophages	18142	**1.** Number of Cells: **36,133** (8 samples)<br>**2.** Total Number of Reads: **2,581,336,274**<br>3. Mean Reads per Cell: **71,440**<br>**Tec: Illumina NovaSeq 6000**
	T cells	7666	
	$SCGB1A1^+$ Epithelial	3903	
	$S100A12^+$ Macrophages	3876	
	Erythroid cells	1387	
	Neutrophils cells	712	
	$EPCAM^+$ Epithelial cells	400	
	Ciliated cells	43	
	Fibroblasts	4	
Rhesus Macaques	$MARCO^+$ Macrophages	22764	1. Number of Cells: **64,762 (6 samples)**<br>2. Total Number of Reads: **1,534,665,114**<br>3. Mean Reads per Cell: **23,697**<br>**Tec: NovaSeq S4**
	$FCGR2B^+$ Macrophages	14181	
	$CD4^+$ T cells	9340	
	$GNLY^+$ T/NK cells	8406	
	$SIGLEC1^+$ Macrophages	4536	
	$GZMB^+$ T/NK cells	4151	
	Granulocytes	643	
	Ciliated cells	281	

	Fibroblasts	276	
	Epithelial cells	184	

Table S3. Viral RNA⁺ cells observed in patients with COVID-19 BALFs and five infected animals BALF and whole lung datasets.

Viral RNA	Mouse		Hamsters		Human		AGM		Ferrets		Macaques	
	#Cells	%	#Cells	%	#Cells	%	#Cells	%	#Cells	%	#Cells	%
Orf10	2878	52.25	1066	6.51	292	0.57	1245	2.72	0	0.00	2	0.003
Orf1ab	2491	45.23	446	2.72	322	0.62	656	1.43	2	0.005	2	0.003
Orf3a	516	9.39	66	0.40	42	0.08	87	0.19	0	0.00	0	0.00
Orf7a	667	12.10	90	0.55	59	0.11	108	0.24	0	0.00	0	0.00
Orf6	534	9.69	91	0.57	25	0.05	45	0.09	0	0.00	0	0.00
Orf8	324	5.88	381	2.34	84	0.17	38	0.08	0	0.00	0	0.00
M	733	13.30	215	1.32	44	0.10	118	0.26	1	0.002	0	0.00
N	2105	38.22	79	0.49	654	1.28	1216	2.66	0	0.00	0	0.00
S	1484	26.94	215	1.31	77	0.15	289	0.63	1	0.002	0	0.00
E	115	2.09	16	0.09	9	0.02	7	0.03	0	0.00	0	0.00
hACE2	76	1.38	0	0.00	5	0.01	0	0.00	0	0.00	0	0.00
sgRNA-N	63	1.14	23	0.14	18	0.03	7	0.03	0	0.00	0	0.00

Definition of abbreviations: AGM = african green monkey; Orf = open reading frame; M = membrane protein; N = nucleoprotein; S = spike; E = envelope protein; hACE2 = human angiotensin-converting enzyme 2; sgRNA-N = subgenomic RNA
